# Natural Inhibitors of *Salmonella* MDR Efflux Pumps AcrAB and AcrD: An Integrated In Silico, Molecular, and In Vitro Investigation

**DOI:** 10.3390/ijms252312949

**Published:** 2024-12-02

**Authors:** Azza S. El-Demerdash, Shimaa A. Kamel, Eman Y. T. Elariny, Hanan Henidi, Yasmin Mahran, Hadil Alahdal, Abdulrahman M. Saleh, Rehab A. Ibrahim

**Affiliations:** 1 Laboratory of Biotechnology, Department of Microbiology, Agricultural Research Center (ARC), Animal Health Research Institute (AHRI), Zagazig 44516, Egypt; 2 Department of Botany and Microbiology, Faculty of Science, Zagazig University, Zagazig 44519, Egypt; shimaa93.ahmed@gmail.com (S.A.K.); eytohamy@zu.edu.eg (E.Y.T.E.); rehabatef@yahoo.com (R.A.I.); 3 Research Department, Natural and Health Sciences Research Center, Princess Nourah bint Abdulrahman University, P.O. Box 84428, Riyadh 11671, Saudi Arabia; hahenidi@pnu.edu.sa; 4 Department of Biology, College of Sciences, Princess Nourah bint Abdulrahman University, Riyadh 13415, Saudi Arabia; hmalahdal@pnu.edu.sa; 5 Department of Pharmaceutical Chemistry, Faculty of Pharmacy, Cairo University, KasrEl-Aini Street, Cairo 11562, Egypt; abdo.saleh240@azhar.edu.eg; 6 Infection Control and Epidemiology Surveillance Unit, Aweash El-Hagar Family Medicine Center, Ministry of Health and Population (MOHP), Mansoura 35711, Egypt

**Keywords:** antimicrobial resistance, efflux pump inhibitors, gene expression, green chemistry, in silico docking, molecular dynamic, sustainable drug development

## Abstract

Multidrug-resistant (MDR) *Salmonella* remains a significant global health threat. This study aimed to explore the potential of essential oil components as novel inhibitors of the *Salmonella* MDR efflux pumps AcrAB and AcrD. *Salmonella* isolates were characterized for serotype, antibiotic resistance, and efflux pump activity. Essential oil components were screened for inhibitory effects using phenotypic and genotypic methods. In silico docking and molecular dynamics simulations were conducted to investigate binding interactions and stability. *Salmonella Typhimurium* was the predominant serotype with high MDR rates. Efflux pump activity was prevalent. Cumin and cinnamon oils demonstrated promising inhibitory effects on these pumps. Molecular docking simulations revealed strong binding affinities of analyzed compounds to the AcrAB and AcrD binding pocket. The 2-methyl-1-(p-tolyl)propan-2-ol exhibited higher stability within the AcrAB binding pocket compared to (1S,3R,5R)-1-isopropyl-4-methylenebicyclo[3.1.0]hexan-3-ol within the AcrD binding pocket. Treatment with these oils significantly downregulated efflux pump genes (*robA*, *acrB*, *mdtB*, *acrF*, *acrD*, *soxS*, *mdsB*, *marA*). The novel approach of combining in silico and molecular dynamics simulations with precise gene expression analysis provides a valuable framework for future studies aimed at combating MDR *Salmonella* efflux pumps.

## 1. Introduction

*Salmonella enterica* serovars are a significant global health concern, causing an estimated three million cases of foodborne illness annually, according to the WHO [1]. The emergence of multidrug-resistant (MDR) *Salmonella* strains has exacerbated this problem, limiting treatment options [2,3,4]. Efflux pumps, which actively expel antibiotics from bacterial cells, are key contributors to MDR. These protein channels embedded in the bacterial cell membrane reduce the intracellular concentration of antibiotics, rendering them less effective [5].

Among the various efflux pump families in *Salmonella*, resistance-nodulation-division (RND) pumps play a pivotal role in multidrug resistance. The AcrAB-TolC complex is the most well-studied RND pump in *Salmonella*. However, another RND pump, AcrD, has also been implicated in conferring multidrug resistance. AcrD is a tripartite complex consisting of an inner membrane efflux pump, a membrane fusion protein, and a TolC-like outer membrane protein. While homologous to the AcrB subunit of the AcrAB-TolC complex, AcrD exhibits distinct substrate specificity and regulatory mechanisms [6,7,8,9]. It is known to confer resistance to a variety of antibiotics, including beta-lactams, fluoroquinolones, and aminoglycosides.

Other efflux pump families in *Salmonella* include major facilitator superfamily (MFS), small multidrug resistance (SMR), and multidrug efflux (MEX) pumps, each with specific substrates [8,9]. Overexpression of efflux pumps can confer resistance to multiple antibiotic classes, making it challenging to treat infections caused by MDR *Salmonella* strains [10]. Understanding the mechanisms of efflux pump-mediated resistance is crucial for developing new strategies to combat these infections.

Plant extracts and natural compounds have demonstrated potential in modulating bacterial resistance, including reversing their inherent resistance to certain antibiotics [11,12,13,14]. Essential oils, concentrated liquids derived from plants, possess various biological properties, such as antimicrobial activity [14,15]. While research on the specific effects of essential oils on efflux pumps in *Salmonella* is limited, their potential to influence bacterial resistance mechanisms warrants further investigation.

This study aimed to uncover the potential of essential oil components as novel inhibitors of the *Salmonella* MDR efflux pumps AcrAB and AcrD. By characterizing *Salmonella* isolates, screening essential oils, gene expression assays, and employing advanced in silico modeling techniques, we sought to identify and investigate compounds that could effectively target these critical resistance mechanisms.

## 2. Results

### 2.1. Salmonella Proportion and Serotype Distribution

**Figure 1:**
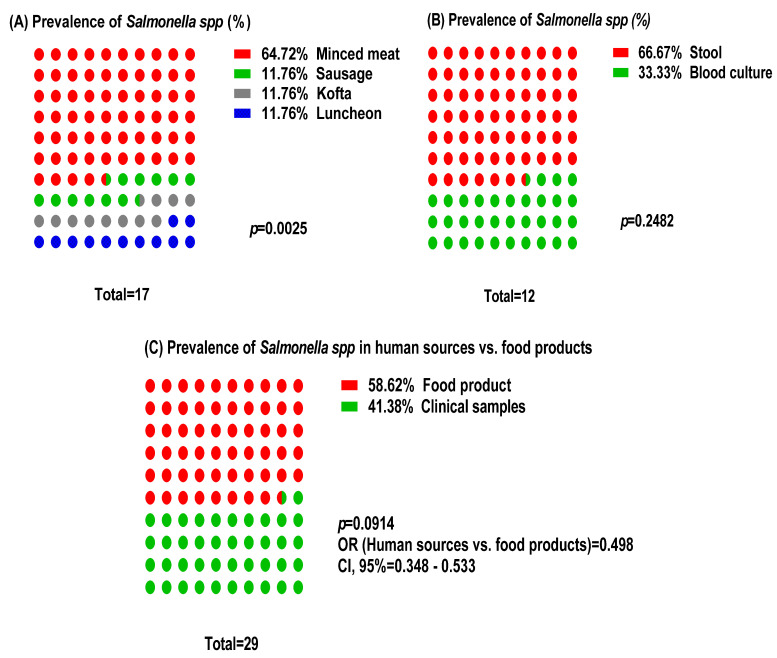
Histogram comparison of *Salmonella* spp. prevalence in food products and human sources.

The prevalence of *Salmonella* spp. was significantly higher in minced meat (n = 11/21, 64.27%) compared to other food products (sausage, kofta, and luncheon meat; 11.76%; *p* = 0.0025; Figure 1A). In human samples, there was no significant difference in *Salmonella* prevalence between stool (66.67%) and blood cultures (33.33%) from patients with gastrointestinal symptoms (*p* = 0.2482; Figure 1B). Overall, *Salmonella* spp. was detected in a higher proportion of food samples (72.41%) compared to human samples (50.20%) (OR = 0.498; *p* = 0.0914; Figure 1C).

**Figure 2:**
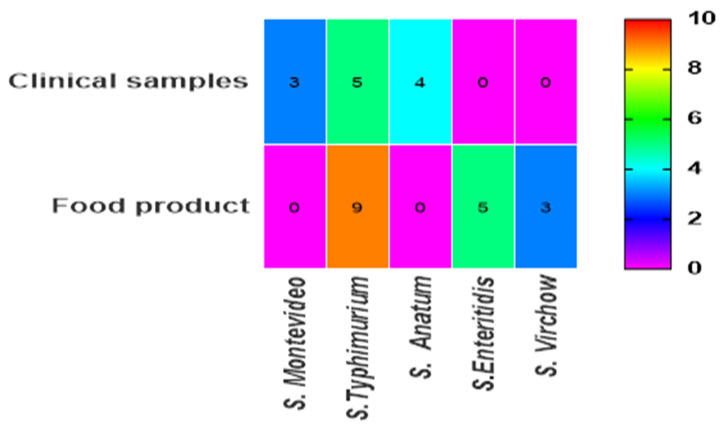
Distribution of *Salmonella* spp. by serotype between two sources.

Regarding serotypes, *S. Typhimurium* was the predominant serotype, with a higher prevalence in food samples (64.29%) compared to human samples (35.71%) (Figure 2). Notably, *S. Enteritidis* and *S. Virchow* were not detected in clinical samples, and *S. Montevideo* and *S. Anatum* were absent from food products.

### 2.2. Antibiotic Susceptibility Pattern and Efflux Pumps

**Figure 3:**
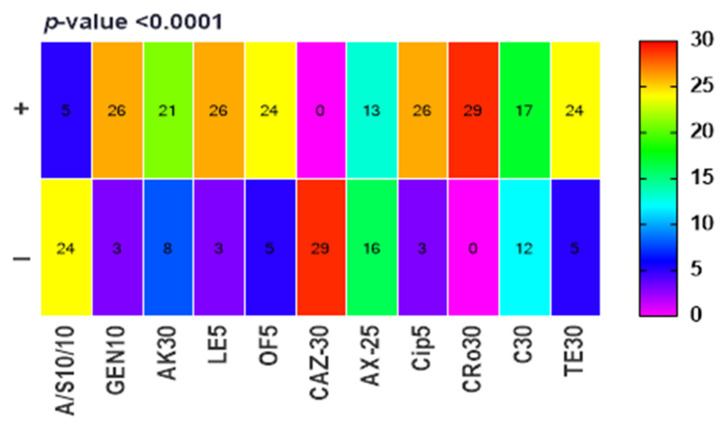
Sensitivity of *Salmonella* spp. to different antibiotics.

*Salmonella* isolates exhibited a high level of susceptibility to the majority of antibiotics tested (*p* < 0.0001). However, resistance was observed to ampicillin/sulbactam, ceftazidime, amoxicillin, and chloramphenicol (Figure 3). This trend was corroborated by the multiple antibiotic resistance (MAR) index, with minced meat isolates demonstrating the highest resistance compared to luncheon meat. Alarmingly, 86.2% (25/29) of isolates displayed a multidrug-resistant (MDR) phenotype (Table 1). Ceftriaxone was the most effective antibiotic among those tested and served as a comparator for subsequent experiments.

The high prevalence of efflux pump activity in 93% of isolates (n = 29) highlights a significant challenge in treating *Salmonella* infections. These efflux pumps likely contribute to the observed antibiotic resistance and MDR phenotype.

### 2.3. Essential Oil Activity

**Figure 4:**
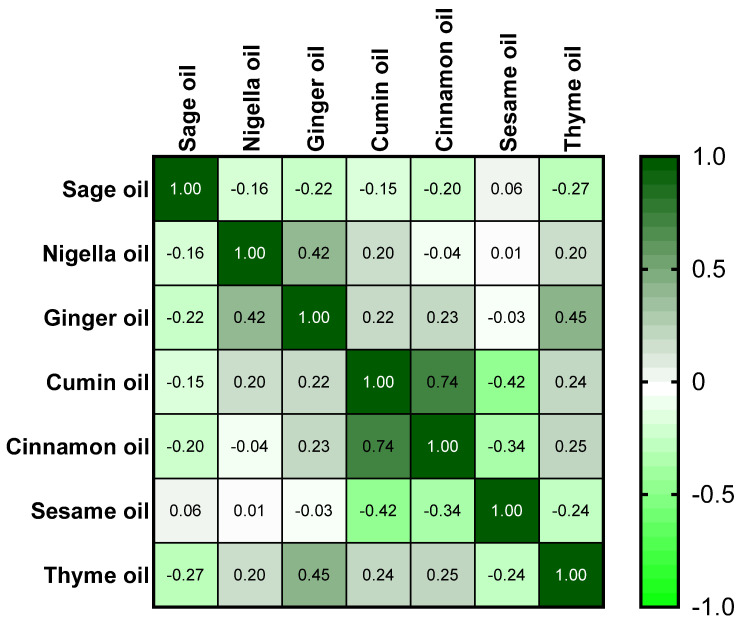
Pearson’s correlation coefficient between inhibition zones (mm) of essential oils against MDR *Salmonella*.

**Figure 5:**
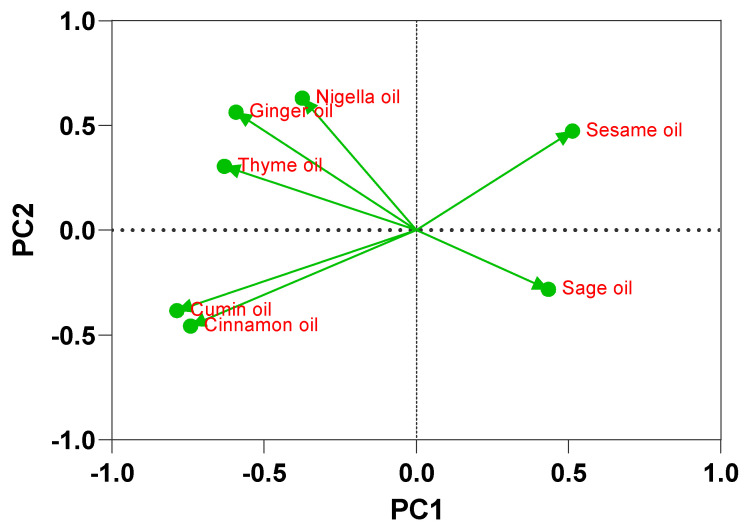
Principal component analysis (PCA) of inhibition zones (mm) of essential oils against MDR *Salmonella* spp.

Cinnamon and cumin oils demonstrated the strongest antimicrobial activity among the tested oils, with inhibition zones ranging from 20 to 36 mm against *Salmonella* isolates through an agar well diffusion test (Figure S17). Principal component analysis (PCA) is a statistical technique used to reduce the dimensionality of data. In the context of essential oil activity, PCA can help to identify patterns and relationships between different oils based on their antimicrobial properties. By analyzing the inhibition zones of various oils against different microorganisms, PCA can group oils with similar antimicrobial profiles. PCA revealed a strong positive correlation between the inhibition zones of Nigella sativa oil and those of cumin and cinnamon oils (r = 0.744; *p* < 0.05, Figure 4). Factor analysis identified four factors that described 80.89% of the total data variability in the essential oil activity. The first dominant factor (35.85% of the total variance) described the combined effects of inhibition zones from ginger, cumin, cinnamon, and thymus oil. The second factor (20.93% of the total variance) revealed significant loading on the properties of Nigella sativa oil. The third component (12.64% of the total variance) gave a strong positive load to the activity of sage oil (Table 2 and Figure 5). PCA and factor analysis can help to optimize the formulation of essential oil-based products for various applications, such as food preservation, personal care, and medicine.

#### Minimum Inhibitory Concentration (MIC) Findings

Both cinnamon and cumin oils exhibited a wide range of antimicrobial activity against the tested *Salmonella* isolates. The minimum inhibitory concentration (MIC) of cinnamon oil ranged from 0.125 to 8 μg/mL, while the minimum bactericidal concentration (MBC) ranged from 0.5 to 32 μg/mL. Similarly, cumin oil displayed an MIC range of 0.125 to 8 μg/mL and an MBC range of 0.5 to 16 μg/mL. Notably, both oils demonstrated sub-inhibitory concentration (SIC) values ranging from 0.125 to 8 μg/mL and 0.125 to 4 μg/mL, respectively, indicating potential effects at concentrations below the MIC (Table S2). The susceptibility range of different isolates to oils can vary based on their genetic makeup and physiological state.

### 2.4. Characterization of Compounds Present in the Oily Extracts (Cinnamon and Cumin) Using GC-MS

GC-MS analysis of cinnamon oil revealed cinnamaldehyde (E)- as the primary constituent, comprising 58.23% of the total composition. Benzaldehyde was identified as the second most abundant component, accounting for 41.61%. Minor components included 1,6-Heptadiyne, benzyl alcohol, formic acid, phenylmethyl ester, N-Cbz-6-Bromo-hexylamine, and 3-Methyl-2-propylcyclopent-2-en-1-one (Table S3, Figure S1).

Similarly, GC-MS analysis of cumin oil identified 39 compounds. The predominant components were 1,4-Cyclohexadiene-1-methanol, 4-(1-methylethyl)- (29.76%), and 2-Caren-10-al (27.95%). Limonene and Eucalyptol were detected as minor constituents (Table S4, Figure S2).

### 2.5. Molecular Docking Study

#### 2.5.1. Molecular Docking of Target Compounds Against Salmonella Typhimurium 

**Figure 6:**
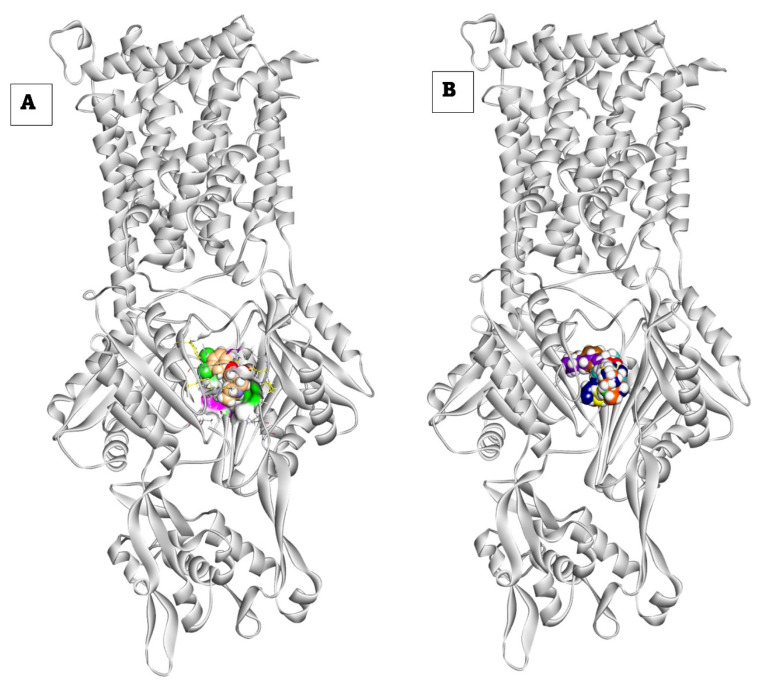
(**A**) 3D figure of the reference ligand (PDB ID: A1AN8) docked inside the target pocket of *Salmonella* MDR efflux pump acrAB. (**B**) 3D figure of the tested compounds occupying the same target site of reference compound inside the target pocket of *Salmonella* MDR efflux pump acrAB.

**Figure 7:**
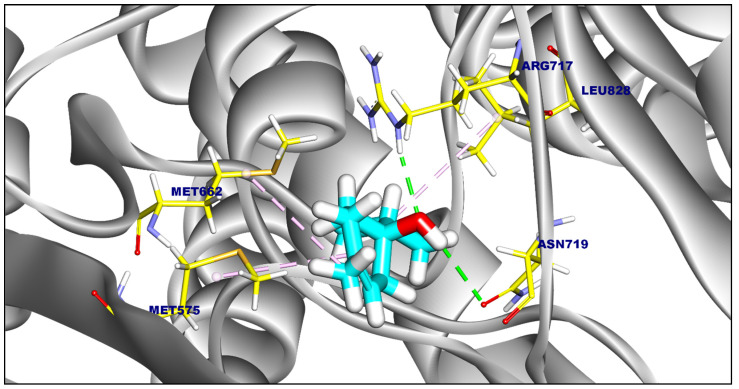
Three-dimensional figure of Compound **22** against *Salmonella Typhi* MDR efflux pump AcrAB.

Proximal Binding Pocket of MDR Efflux Pump AcrAB. All analyzed compounds demonstrated binding affinities to the Salmonella Typhimurium MDR efflux pump AcrAB, as shown in Table 3. These compounds exhibited a binding pattern similar to the reference drug (PDB ID: A1AN8), as illustrated in Figure 6. Compound **12** interacted with Leu828, Ala618, Phe617, Met575, and Phe646 by nine hydrophobic π-interactions and formed one hydrogen bond with Arg717 (Figure S3). Compound **21** formed five hydrophobic π-interactions and one hydrogen bond with Arg717 with a distance of 2.34 Å (Figure S4). Compound **22** interacted by three hydrophobic π-interactions and two hydrogen bonds with Arg717 and Asn719 with distances of 2.43 and 2.69 Å (Figure 7). Compound **23** interacted by four hydrophobic π-interactions with Ala618, Phe664, Met575, and the interaction was supported by two hydrogen bonds with Ala618, and Phe617 with distances of 2.17 and 2.45 Å (Figure S5).

**Figure 8:**
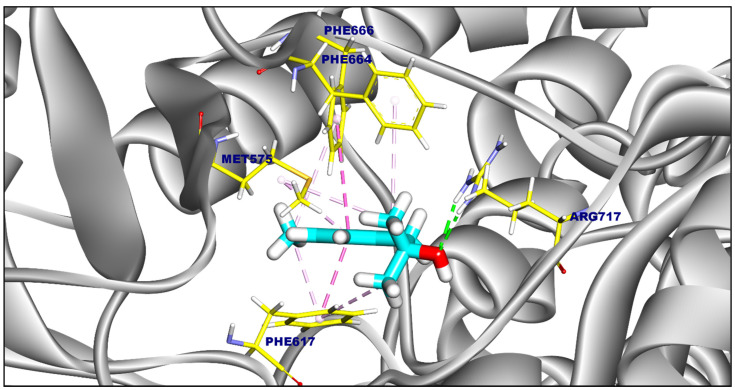
Three-dimensional figure of Compound **24** against *Salmonella Typhi* MDR efflux pump AcrAB.

Compound **24** exhibited the strongest binding affinity (−6.98 kcal/mol), forming eight π-interactions and one hydrogen bond with Arg717 (2.39 Å) (Figure 8). Compound **31** (ΔG = −6.56 kcal/mol) formed four π-interactions and one hydrogen bond with Arg717 (2.55 Å) (Figures S6 and S16). Compound **37** (ΔG = −5.87 kcal/mol) formed seven π-interactions with Leu828, Arg717, Ala618, Leu721, and Met575 (Figure S7).

**Figure 9:**
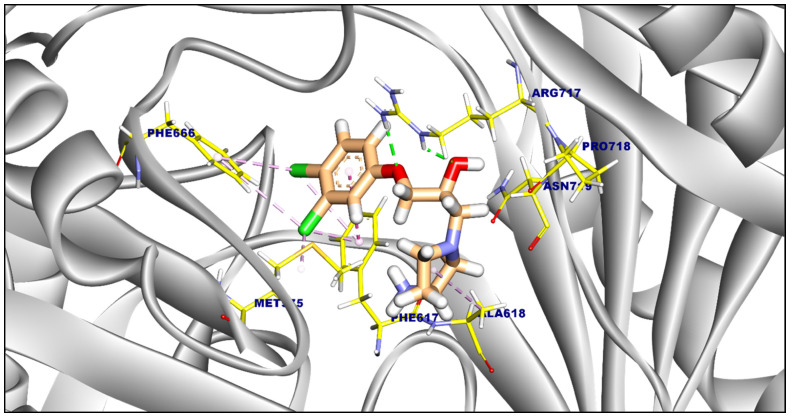
Three-dimensional figure of the reference ligand (PDB ID: A1AN8) against *Salmonella* MDR efflux pump acrAB.

The reference compound (PDB ID: A1AN8) exhibited a binding affinity of −6.88 kcal/mol. It formed seven hydrophobic π-interactions with Ala618, Phe617, Met575, and Phe666, and two hydrogen bonds with Arg717 (2.17 and 2.29 Å) (Figure 9).

#### 2.5.2. Molecular Docking of Target Compounds Against *Salmonella Typhimurium* Proximal Binding Pocket of MDR Efflux Pump AcrD

The binding mode of Compound **4** exhibited a binding energy of −5.56 kcal/mol. against *Salmonella* efflux pump target site. two hydrophobic π-interactions were observed with Glu239. Additionally, Compound **4** formed a hydrogen bond with Thr94 with a bond length of 2.18 Å (Figure S8). Moreover, Compound **12** exhibited an affinity score of −5.36 kcal/ mol, against *Salmonella* efflux pump target site. It interacted with Asn14, and Tyr241 by one hydrogen bond and one hydrophobic π-interaction with a bond length of 2.01 Å (Figure S9). The binding mode of Compound **13** exhibited an affinity score of −6.23 kcal/ mol. against *Salmonella* efflux pump target site. Compound **13** showed three hydrophobic π-interactions with Thr12, Tyr144, and Tyr241 (Figure S10). The binding mode of Compound **16** exhibited an energy binding of −5.61 kcal/ mol. against *Salmonella* efflux pump target site. It formed three hydrophobic π-interactions with Tyr241, Gln91, and Leu135, moreover formed two hydrogen bonds with Ser149, and Tyr241 with bond lengths of 2.15, and 1.98 Å (Figure S11).

**Figure 10:**
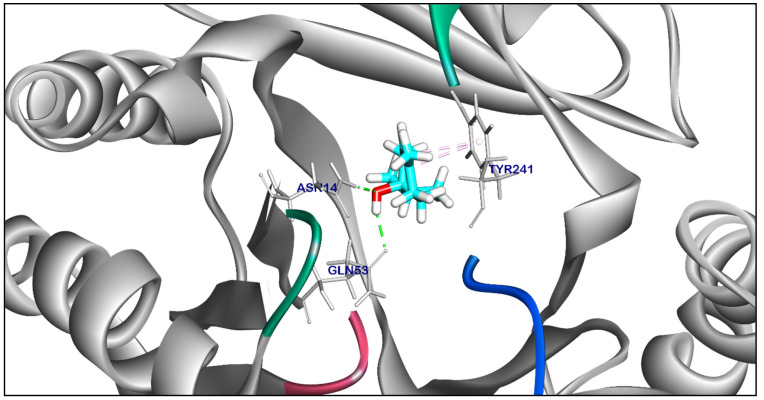
Three-dimensional orientation and of Compound **22** against *Salmonella* efflux pump AcrD target site.

**Figure 11:**
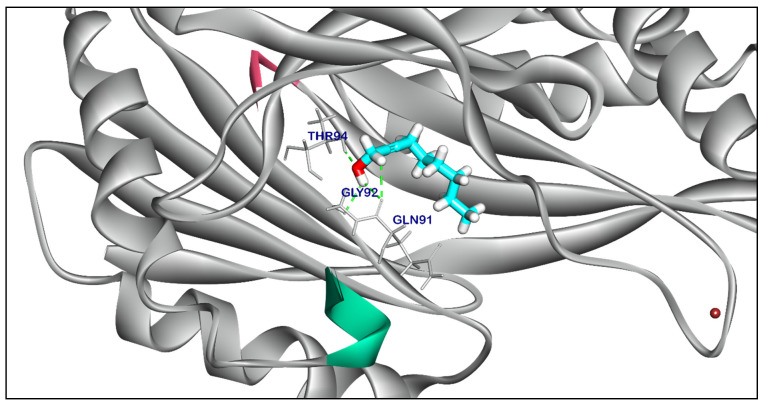
Three-dimensional orientation and of Compound **21** against *Salmonella* efflux pump AcrD target site.

**Figure 12:**
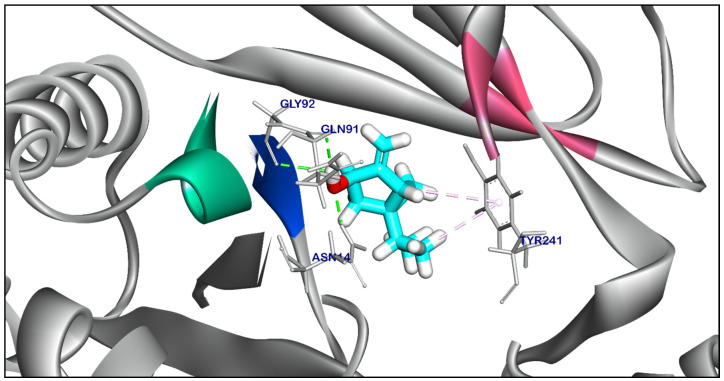
Three-dimensional orientation and of Compound **25** against *Salmonella* efflux pump AcrD target site.

The binding mode of Compound **18** exhibited a binding energy of −7.29 kcal/ mol. against *Salmonella* efflux pump target site. Compound **18** formed two ionic interactions and one hydrophobic π-interactions with Glu289, Arg151, and Gly92. Moreover, Compound **18** interacted with Thr94 by one hydrogen bond with bond lengths of 2.22 Å (Figure S12). The binding mode of Compound **22** exhibited an energy binding of −5.41 kcal/ mol. against *Salmonella* efflux pump target site. Compound **22** formed two hydrophobic π-interactions with Tyr241, and formed two hydrogen bonds with Asn41, and Gln53 with distances of 2.15, and 2.36 Å (Figure 10). The binding mode of Compound **21** exhibited an affinity score of −6.21 kcal/ mol. against *Salmonella* efflux pump target site. Compound **21** formed a hydrophobic π-interaction with Glu91, additionally, three hydrogen bonds were obtained by interaction with Thr94, Gly92, and Gln91 with a distance of 2.04, 2.05, and 2.13 Å (Figure 11). The binding mode of Compound **23** and Compound **24** exhibited affinity scores of −5.23 and −6.27 kcal/ mol., respectively against *Salmonella* efflux pump target site. Compound **23** formed two hydrophobic interactions with Tyr241, and one hydrogen bond with Arg151 with a distance of 1.98 Å (Figure S13). while Compound **24** formed two hydrophobic interactions with Tyr144, and Tyr241, and additionally, interacted with Asn197 by one hydrogen bond with a bond length of 2.06 Å (Figure S14). The binding mode of Compound **25** exhibited a high affinity score equal −7.64 kcal/ mol. against *Salmonella* efflux pump target site. Compound **25** formed two hydrophobic π-interaction with Tyr241. Additionally, interacted with Gly92, Gln91, and Asn14 by three hydrogen bonds with bond lengths of 2.61, 2.11, and 2.54 Å (Figure 12). While Compound **29** exhibited ∆G score equal −5.87 kcal/ mol. against *Salmonella* efflux pump target site. Compound **29** formed two hydrophobic π-interactions with Tye144, and Tyr241, moreover interacted with Tyr241 by a hydrogen bond with a distance of 2.01 Å (Figures S14–S15).

### 2.6. Molecular Dynamic (MD) Simulation Study

**Figure 13:**
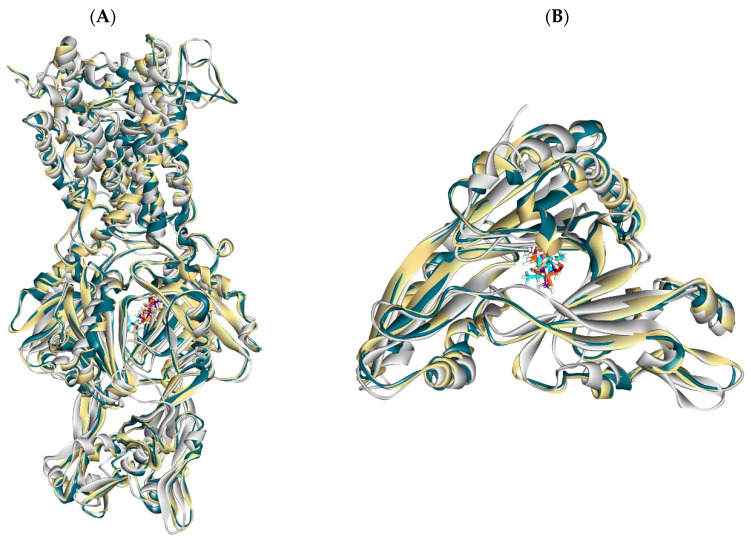
(**A**) 3D figure of Compound **24** against *Salmonella* MDR efflux pump AcrAB. (**B**) 3D figure of Compound **25** against *Salmonella* MDR efflux pump AcrD at 0 ns blue color, 50 ns yellow color, and 100 ns gray color.

To investigate the stability and dynamics of the protein–ligand complexes, molecular dynamics (MD) simulations were conducted for 100 ns. The root mean square deviations (RMSDs) for the complexes and ligands were calculated to assess their conformational stability within the active site. Frontier compound interactions were also analyzed in detail. Additionally, the MM-GBSA free binding energy was estimated for each complex during the simulation trajectories and 3D frames of all changes that occurred in the complexes during the simulation period were also presented in Figure 13.

#### 2.6.1. Protein and Ligand RMSD and RMSF Analysis

**Figure 14:**
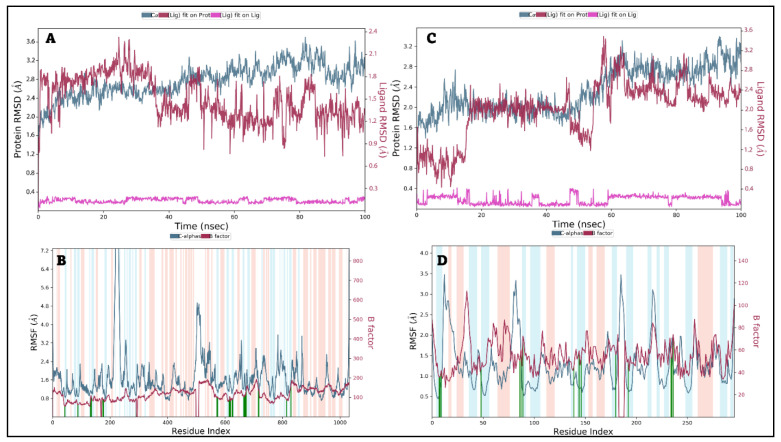
Root mean square deviation (RMSD) and root mean square fluctuation (RMSF) analysis of Compound **24** with AcrAB and Compound **25** with AcrD in *Salmonella Typhimurium*; (**A**,**B**) RMSD and RMSF plots for Compound **24** bound to AcrAB of *Salmonella Typhimurium*, simulated for 100 ns. (**C**,**D**) RMSD and RMSF plots for Compound **25** bound to AcrD of *Salmonella Typhimurium*, simulated for 100 ns.

Compounds 24/AcrAB and 25/AcrD complexes were selected for MD simulation. The conformational stability of the protein structures was monitored through the C*α* atoms of the protein concerning their initial position. As shown in Figure 14A, Compound **24**/AcrAB complex showed high stability inside the target pocket with an RMSD value within 1.80 Å, which is an acceptable value below 3.00 Å. Compound **24** showed stability between 0–40 ns then a minor fluctuation occurs at 45–55 ns and showed stability until the end of simulation time with minor fluctuation at 75–77 ns. Additionally, the protein structure of AcrAB showed notable stability over the simulation time and fluctuated within 1.50 Å. Minor fluctuations were observed in the amino acid regions 230–250 and 500–550, indicating subtle conformational changes. However, these changes did not significantly impact ligand binding within the active site (Figure 14B). On the other hand, Compound **25**/AcrD complex showed some major fluctuations during simulation time at 15–20 ns and the period form 55–65 ns that proves the area of amino acids ligand interaction had many conformational changes, the ligand simulation fluctuated within 2.5 Å and the protein skeleton fluctuated within 1.7 Å that indicate some sort of stability of ligand inside the target pocket sometimes of the simulation run (Figure 14C). The AcrD protein structure showed many fluctuations around the 10–30, 80–90, 180–190, and 225–230 amino acids area, which can affect the binding pattern of Compound **25** with the AcrD binding site (Figure 14D).

#### 2.6.2. Protein–Ligand Interactions Analysis

##### Histogram of Protein–Ligand Interactions Analysis

**Figure 15:**
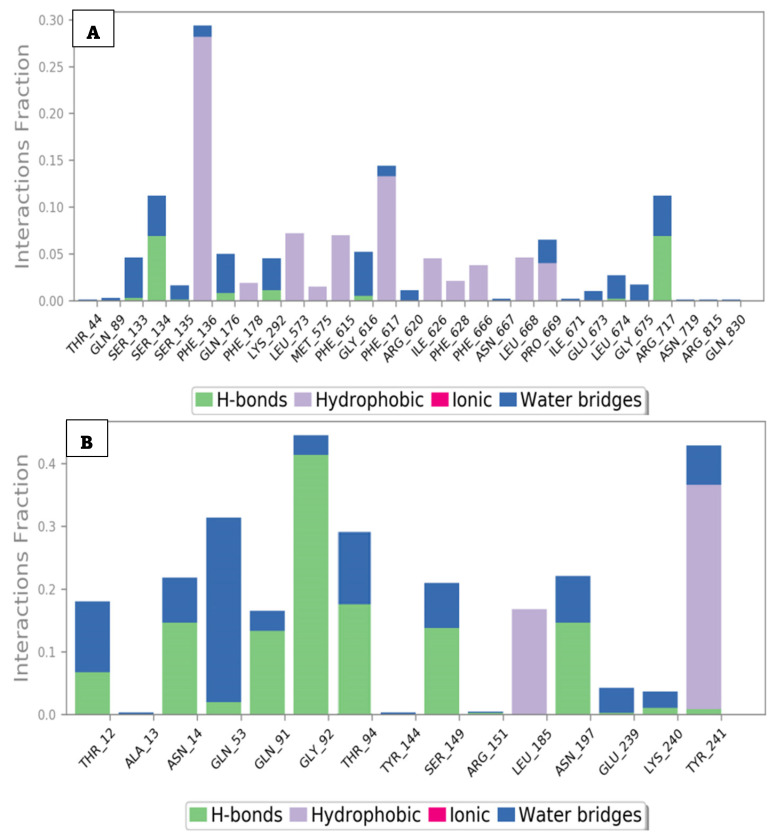
Histogram analysis describing the binding interactions of Compounds 24 (**A**) and 25 (**B**) with MDR efflux pump AcrAB and AcrD of *Salmonella* during the simulation time (100 ns).

The target compounds showed higher stability with AcrAB when compared to the interactions with AcrD (Table 4). Compound **24** formed H-bond interactions with the following residues: Ser134 (~7.5%), Gln176-Lys292-Gly616 (~5%), and Arg717 (~10%), as presented in Figure 15A. Compound **25** interacted with MDR efflux pump AcrD by many hydrogen bonds with residues, Thr12 (~7%), Asn14 (~15%), Gln53 (~2%), Gln91 (~15%), Gly92 (~45%), Thr94 (~20%), Ser149 (~15%), and Asn197 (~15%).

Compound **24** formed water-bridged H-bonds with residues Ser133 (~5%), Ser134 (~10%), Gln176 (~5%), Lys292 (~5%), Gly616 (~5%), Pro669 (~3%), Leu674 (~10%), and Arg717 (~10%) (Figure 14A). Compound **25** formed water-bridged H-bonds with residues Thr12 (~10%), Asn14 (~5%), Gln53 (~30%), Thr94 (~15%), Ser149 (~5%), Asn197 (~5%), Glu239 (~5%), and Tyr241 (5%) (Figure 15B). Additionally, Compound **24** formed hydrophobic interactions with residues Phe136 (~30%), Leu573 (~10%), Met575 (~10%), Phe617 (~15%), Ile626 (~5%), Phe666 (~5%), Leu668 (~5%), and Pro669 (~5%). Compound **25** formed hydrophobic interactions with residues Leu185 (~20%) and Tyr241 (~40%).

**Figure 16:**
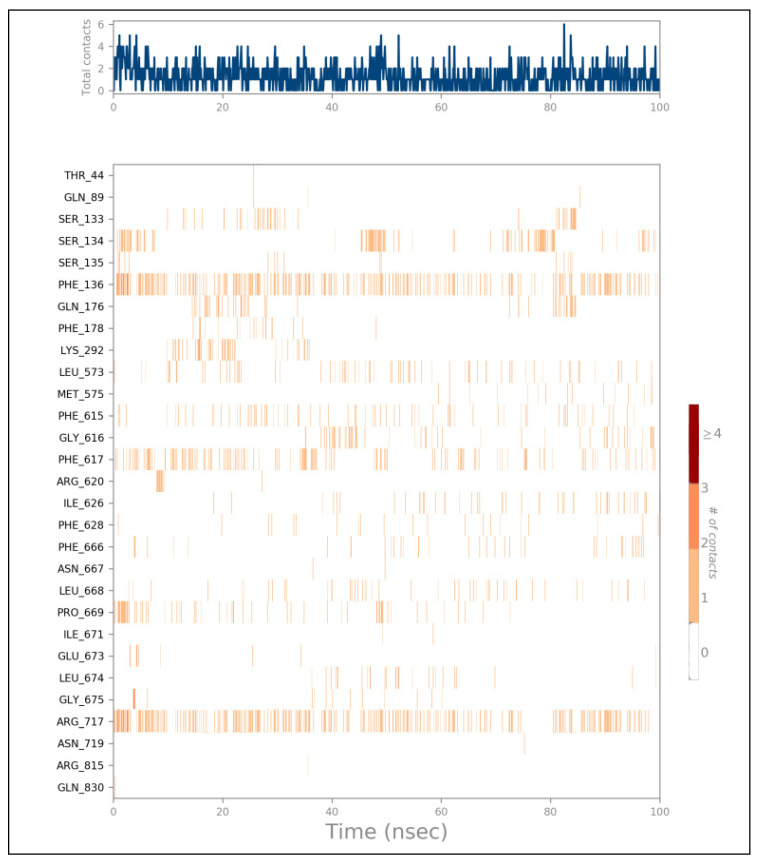
Heat map describing the total number of interactions within Compounds 24 with MDR efflux pump AcrAB of *Salmonella* complex during the 100 ns.

**Figure 17:**
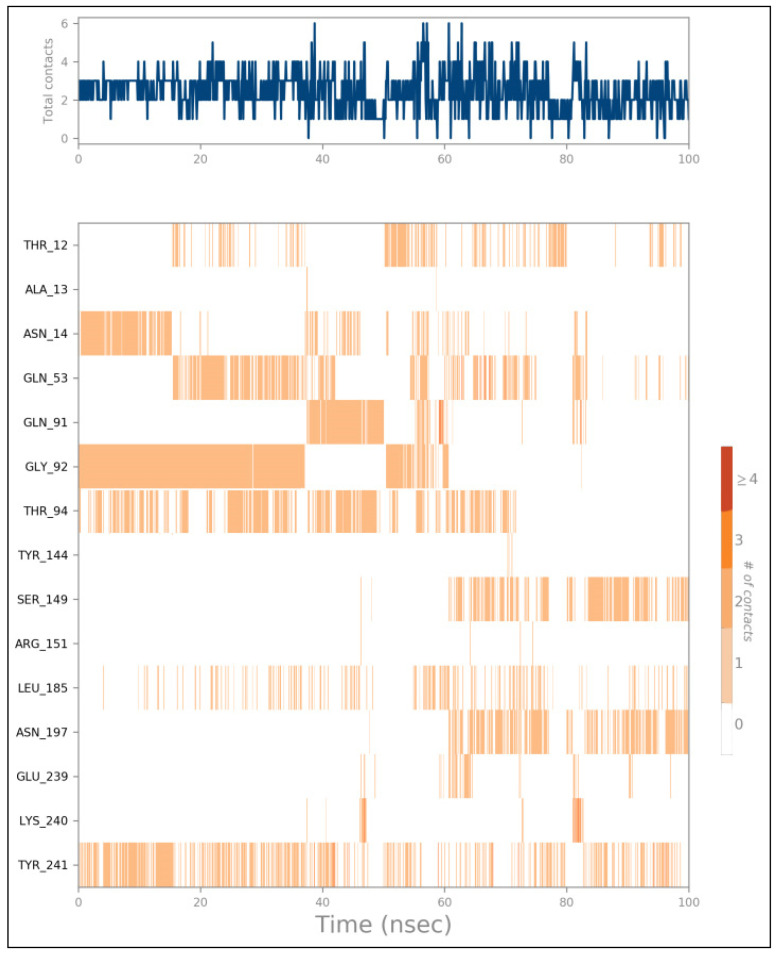
Heat map describing the total number of interactions within Compounds 25 with MDR efflux pump AcrD of *Salmonella* complex during the 100 ns.

To analyze the dynamic nature of the protein–ligand interactions, heat maps were generated to visualize the number of interactions over time (Figure 16 and Figure 17). The heat maps revealed that both Compound **24**/AcrAB and Compound **25**/AcrD complexes formed up to four hydrogen bonds during the simulations. Key amino acid residues involved in the interactions with Compound **24** included Phe136, Phe617, and Arg717. Compound **25** interacted with Asn14, Gln53, Gly92, Thr94, Ser149, Tyr241, and Leu145.

##### MM-GBSA Calculations

**Figure 18:**
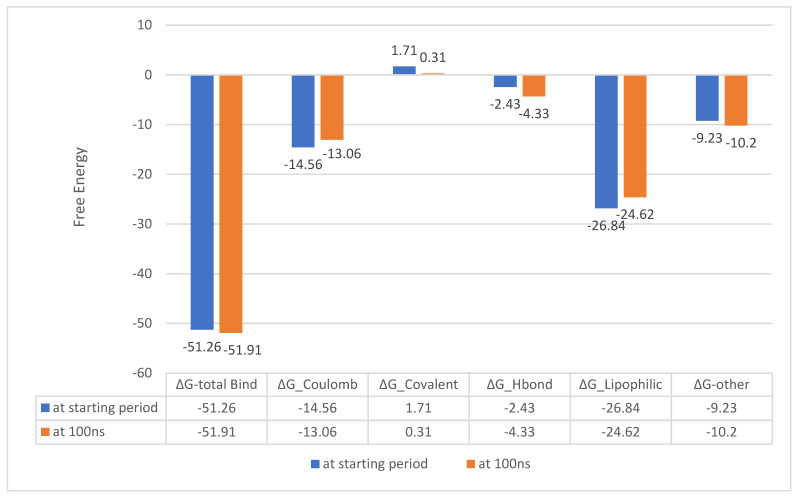
MM-GBSA energies for (Compounds 25 with MDR efflux pump AcrD of *Salmonella Typhimurium* (kcal/mol).

**Figure 19:**
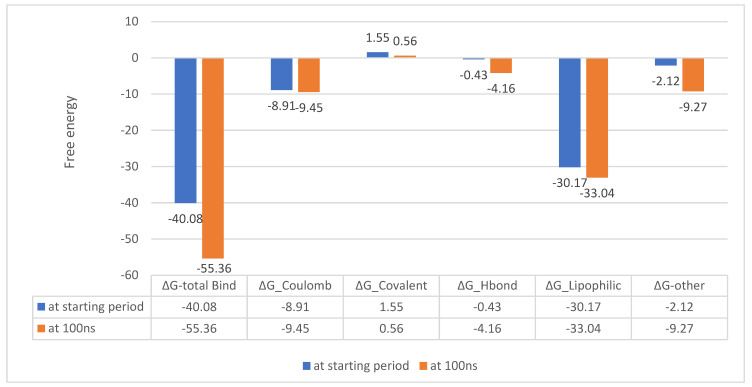
MM-GBSA energies for (Compounds 24 with MDR efflux pump AcrAB of *Salmonella Typhimurium* (kcal/mol).

**Figure 20:**
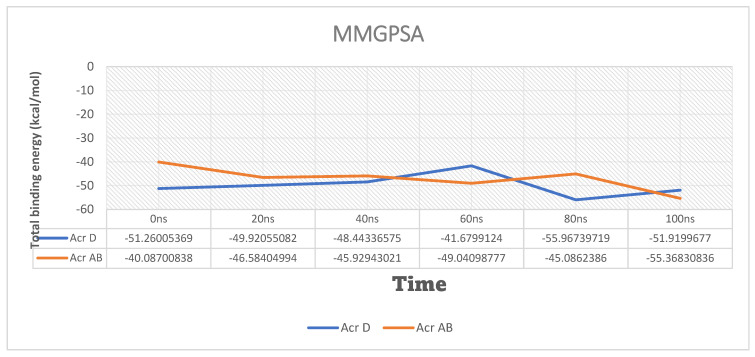
Total MM-GBSA energies for (Compounds 24 with MDR efflux pump AcrAB and Compounds 25 with MDR efflux pump AcrD of *Salmonella Typhimurium* (kcal/mol).

Molecular mechanics generalized born surface area (MM-GBSA) calculations were performed to estimate the binding free energies of the compounds with the AcrAB and AcrD complexes. The results indicate that Compound **25**/AcrD exhibited a stable binding interaction throughout the simulation (Figure 18), while Compound **24**/AcrAB showed a notable increase in free energy at the end of the simulation, suggesting a less stable interaction (Figure 19). From the next results, Compound **25**/AcrD showed no change in ∆G from the starting period until the end of the simulation time, while Compound **24**/AcrAB showed a notable increase in free energy at the end of the simulation time rather than the starting point, which indicates Compound **24**/AcrAB has more stability at the end of simulation process (Figure 20).

### 2.7. Detection of Efflux Pump Genes by Conventional PCR

Efflux pump genes were detected in a high proportion of *Salmonella* isolates, with *robA*, *AcrB*, *mdtB*, *acrF*, and *acrD* being the most prevalent, with detection rates ranging from 72.4% to 93%. The presence of multiple efflux pump genes in many isolates suggests a complex mechanism of antibiotic resistance. Isolates harboring a greater number of efflux pump genes generally exhibited higher levels of multidrug resistance (Table 1).

### 2.8. Modulatory Effects of Cumin and Cinnamon Oils on Efflux Pump Gene Transcription in Salmonella

**Figure 21:**
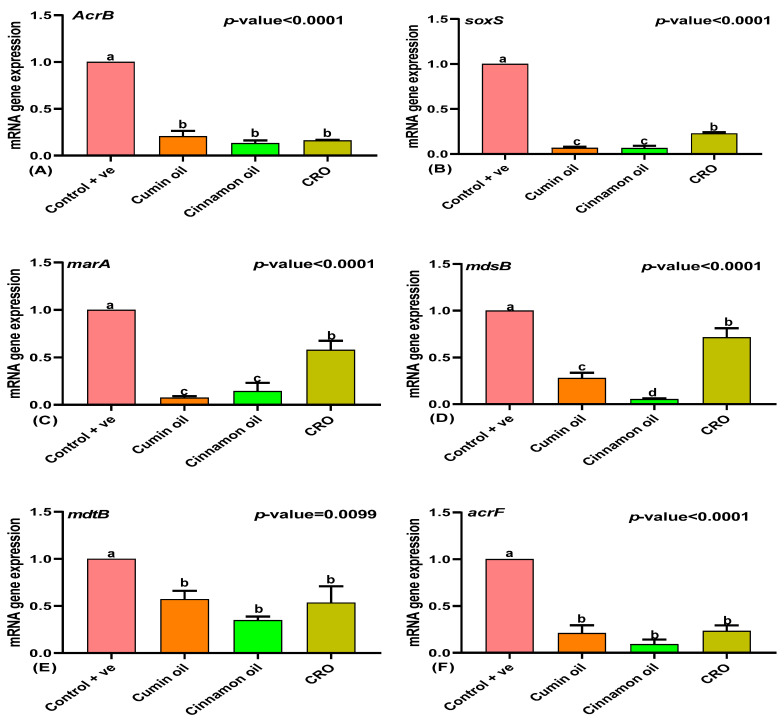

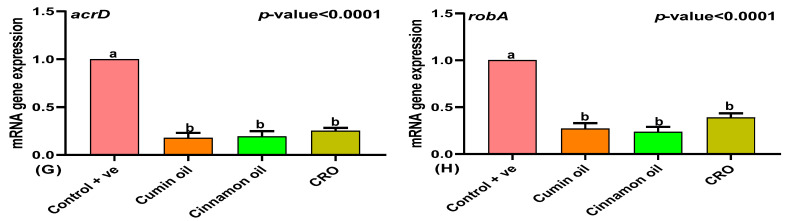
Modulatory effect of different treatments on efflux pump-related genes (**A**) *acrB*, (**B**) *soxS*, (**C**) *marA*, (**D**) *mdsB*, (**E**) *mdtB*, (**F**) *acrF*, (**G**) *acrD*, and (**H**) *robA*. Different letters (a,b,c,d) indicate significant changes.

Treatment with sub-inhibitory concentrations (SICs) of cumin and cinnamon oils significantly downregulated the expression of efflux pump genes (*acrB*, *mdtB*, *acrF*, and *acrD*) in *Salmonella* compared to the untreated control group (*p* < 0.05; Figure 21). Although there was no significant difference in the expression of the regulatory genes *soxS*, *marA*, and *robA* between the cumin and cinnamon oil groups (*p* > 0.05), both oils effectively reduced their expression compared to both the control and ceftriaxone (CRO)-treated groups (*p* < 0.05; Figure 21B,C,G,H). Notably, cinnamon oil exhibited a more pronounced inhibitory effect on *mdsB* transcription compared to cumin oil (*p* < 0.05; Figure 21D). These results suggest that both cumin and cinnamon oils can effectively inhibit efflux pump activity in *Salmonella* by downregulating the expression of key efflux pump genes and their regulatory factors.

## 3. Discussion

The emergence of multidrug-resistant (MDR) *Salmonella* strains poses a significant public health challenge. This study investigated the prevalence of *Salmonella* in food and human samples, characterized their antibiotic resistance profiles, and explored the potential of plant-derived compounds to mitigate these issues.

Our findings revealed a higher prevalence of *Salmonella* in food products compared to human samples, which aligns with previous studies [16,17,18]. The lower prevalence of *Salmonella* spp. in human samples compared to food products suggests that effective food safety measures are being implemented at certain points in the food chain. However, the high prevalence of minced meat highlights a potential source of contamination. The absence of specific serotypes (*S. Enteritidis* and *S. Virchow*) in clinical samples or food products could be attributed to several factors, including regional variations in circulating serotypes, differences in surveillance strategies, or the impact of control measures. The dominance of *S*. *Typhimurium* in both food and human samples suggests its significant role in *Salmonella* infections in the region.

The observed antibiotic resistance patterns indicate a concerning trend of multidrug resistance among *Salmonella* isolates. The high levels of resistance to ampicillin/sulbactam, azithromycin, and chloramphenicol highlight the need for continued surveillance and the development of alternative therapeutic strategies. These findings differ slightly from previous studies [19,20], highlighting the geographical variation in antibiotic resistance patterns and emphasizing the need for continued monitoring of antibiotic resistance patterns in *Salmonella*.

The high MAR index, particularly in isolates from minced meat (MAR index = 0.8), suggests a worrying trend of multidrug resistance (MDR) in *Salmonella*. This complicates treatment options, as infections caused by MDR *Salmonella* become more challenging to eradicate. The observation of 86.2% of isolates displaying an MDR pattern further reinforces this concern and great hazard to public health.

The high prevalence of antibiotic resistance, particularly among isolates from minced meat, is concerning. The emergence of MDR strains, as evidenced by the MAR index and the resistance profiles, underscores the need for alternative treatment strategies.

The fact that ceftriaxone remained the most effective antibiotic highlights its current importance in treating *Salmonella* infections. However, continued reliance on a single antibiotic can lead to the emergence of resistance in the future.

The concerning finding of 93% of isolates exhibiting efflux pump activity highlights a significant challenge in treating *Salmonella* infections. Efflux pumps can reduce the effectiveness of antibiotics rendering them less potent.

The combination of MDR and efflux pump activity poses a significant challenge for effectively treating *Salmonella* infections. This necessitates continued research and development of alternative therapeutic strategies to combat these evolving resistance mechanisms. Additionally, efflux pumps play a crucial role in facilitating antibiotic resistance by actively expelling antibiotics from the bacterial cell. Inhibition of these efflux pumps using EOs can increase the intracellular concentration of antibiotics, enhancing their efficacy against MDR *Salmonella* strains [21].

Cumin and cinnamon oils demonstrated promising antibacterial activity and the ability to downregulate the expression of key efflux pump genes, including *robA*, *acrB*, *mdtB*, *acrF*, *acrD*, *soxS*, and *marA. AcrB*, the central component, is responsible for drug recognition and efflux [22]. The *mdtB* efflux pump, belonging to the major facilitator superfamily, contributes to resistance to a broad spectrum of antibiotics [23]. *AcrF*, another member of the RND family, plays a role in multidrug resistance in *Salmonella* [24].

The *robA* gene encodes a transcriptional regulator that plays a crucial role in the expression of efflux pump genes in *Salmonella* [25]. *robA* acts as a transcriptional repressor, downregulating the expression of efflux pump genes under specific conditions. By binding to specific promoter regions of efflux pump genes, *robA* physically hinders the binding of RNA polymerase, thereby preventing transcription initiation. This regulatory mechanism effectively limits the activity of efflux pumps, reducing the efflux of antibiotics and other toxic substances from the bacterial cell [26,27]. Designing of novel primer targeting this gene was impactful and significant.

The regulatory genes *soxS* and *marA* control the expression of efflux pump genes in response to environmental stresses, including antibiotic exposure. *SoxS* regulates the expression of the AcrAB-TolC system, while *marA* controls a wider range of efflux pumps and outer membrane proteins [28]. The *mdsB* gene encodes a component of the minor facilitator superfamily efflux pump, contributing to the overall efflux capacity of the cell.

Downregulation of these efflux pump genes by cumin and cinnamon oils suggests a potential mechanism for their antibacterial activity. By inhibiting the expression of these genes, these natural compounds may increase the intracellular concentration of antibiotics, enhancing their efficacy and overcoming antibiotic resistance.

GC-MS analysis identified cinnamaldehyde and benzaldehyde as the primary constituents of cinnamon oil aligns well with previous studies on the chemical composition of cinnamon essential oil. These compounds have been extensively studied for their antimicrobial properties and are known to contribute significantly to the overall antimicrobial activity of cinnamon oil [29,30]. Similarly, the identification of 1,4-Cyclohexadiene-1-methanol and 2-Caren-10-al as major components of cumin oil is consistent with previous research. These compounds have also been reported to possess antimicrobial activity, supporting the observed inhibitory effects of cumin oil against *Salmonella* [31,32].

Molecular docking simulations demonstrated that all analyzed compounds exhibited strong binding affinities to the proximal binding pocket of the *Salmonella* Typhimurium MDR efflux pump AcrAB, suggesting their potential as inhibitors. Compound **24** exhibited higher stability within the AcrAB binding pocket compared to Compound **25** within the AcrD binding pocket.

The conformational changes observed in the acrD protein structure suggest that the binding of Compound **25** may be influenced by the flexibility of the AcrD protein. Further analysis is needed to understand the implications of these conformational changes on the binding affinity and efficacy of Compound **25** as an inhibitor of the AcrD efflux pump.

The results indicate that Compound **24** exhibits a higher affinity for the AcrAB efflux pump compared to AcrD. This suggests that Compound **24** may be more effective in inhibiting the AcrAB pump, which could potentially be beneficial in combating multidrug-resistant *Salmonella* infections.

The identification of specific amino acid residues involved in the binding interactions between the compounds and AcrAB provides novel and valuable insights into the mechanism of action of these compounds. This information may inform the design of future inhibitors targeting the AcrAB efflux pump.

Both Compound **24** and Compound **25** form a variety of interactions with the MDR efflux pump AcrAB, including water-bridged hydrogen bonds and hydrophobic interactions. These interactions contribute to the binding affinity of the compounds and their potential inhibitory effects on the efflux pump.

The heat maps provide valuable insights into the dynamics of the protein–ligand interactions over time. The identification of the most frequently interacting amino acid residues in each complex can help to elucidate the key determinants of binding affinity and specificity.

The MM-GBSA calculations revealed that Compound **25**/AcrD showed no change in ΔG from the starting period until the end of simulation time, indicating a stable binding interaction. In contrast, Compound **24**/AcrAB showed a notable increase in free energy at the end of the simulation time, suggesting that the binding stability of Compound **24** with AcrAB may decrease over time.

These findings suggest that Compound **25** may have a more stable interaction with the AcrD efflux pump compared to Compound **24** with the AcrAB efflux pump.

## 4. Materials and Methods

### 4.1. Sampling Methodology

A total of 100 samples were randomly collected for the study: 61 food samples and 39 human samples. Food samples included 21 minced meat, 27 sausage, 4 kofta, and 9 luncheon meat, collected from supermarkets and butcher shops in Al Sharkia Governorate, Egypt, between 2022 and 2024. Human samples (21 stool and 18 blood) were obtained from patients with gastroenteritis symptoms in hospitals and clinical laboratories in the same region during the same period. Human patients were selected based on their reported symptoms. Food samples were collected using aseptic techniques.

### 4.2. Isolation and Identification

Standard cultivation methods, as recommended by ISO 6579-1: 2017 [33], were employed with modifications. Samples were homogenized at 10% in buffered peptone solution using a Stomacher 400R (Seward, London, UK) and incubated overnight at 37 °C. Pre-enrichment was performed in Rappaport Vassiliadis (RV) broth (Lab M, Bury, UK) at 41 °C for 18–24 h. *Salmonella* was isolated on XLD agar plates (BioLife, Bothell, Washington, DC, USA) at 37 °C for 18–24 h. Atypical colonies were further confirmed on MSRV, HE, and Bismuth Sulfite Agar. Suspect colonies were biochemically characterized using the API 20E^®^ system (BioMerieux, Marcy-l’Étoile, France). Blood samples were enriched using bile salt broth and streptokinase broth according to Nagshetty et al. [34] and subsequently cultured on selective agar.

Serological identification of somatic (O) and flagellar (H) antigens was performed on biochemically confirmed smooth isolates using the Kauffman–White scheme. Rough autoagglutinable isolates were excluded from serotyping.

### 4.3. Antibiotics Susceptibility Testing

Antimicrobial susceptibility testing was performed using the Kirby–Bauer disk diffusion method [35] on Mueller–Hinton agar according to CLSI guidelines [36]. Bacterial suspensions were prepared to a 0.5 McFarland standard. Antimicrobial disks containing Ampicillin/Sulbactam (A/S/10/10), gentamicin (CN, 10 μg), amikacin (AK, 30 μg), levofloxacin (LE, 5 μg), ofloxacin (OF, 5 μg), ceftazidime (CAZ, 30 μg), amoxicillin (AX, 25 μg), ciprofloxacin (CIP, 5 μg), ceftriaxone (CRO, 30 μg), chloramphenicol (C, 30 μg), and tetracycline (TE, 30 μg) were applied to inoculated plates. Zones of inhibition were measured and interpreted according to CLSI standards. Quality control strains were included in each testing run to ensure accurate results.

The multiple antibiotic resistance (MAR) index was calculated as the ratio of resistant antibiotics to total antibiotics tested [37]. This index is used to assess the overall level of antibiotic resistance in a bacterial population. Isolates resistant to at least three different antibiotic classes were considered multidrug-resistant [38]. The commercial strain ATCC^TM^ 14028 was used as a reference and comparative phenotype.

### 4.4. Essential Oils (EOs)

Seven essential oils (98% purity) were obtained from the Medicinal and Aromatic Oils Unit at the National Research Center, Doki, Egypt. The oils included:

Oil NameBotanical NameSage oil
*Salvia officinalis*
Nigella oil
*Nigella sativa*
Ginger oil
*Zingiber officinale*
Cumin oil*Cuminum cyminums* L.
Cinnamon oil
*Cinnamomum verum*
Sesame oil
*Sesamum indicum*
Thyme oil
*Thymus vulgaris*


### 4.5. Agar Well Diffusion Assay of Herbal Oils

The antibacterial activity of seven essential oils against *Salmonella* isolates was investigated using the agar well diffusion method. Bacteria were cultured in nutrient broth at 37 °C for 24 h and adjusted to a concentration of 1.5 × 10^8^ CFU/mL. Mueller–Hinton agar plates were inoculated with bacterial suspensions.

A volume of 200 µL of each essential oil was selected to ensure sufficient oil for diffusion and reliable zone of inhibition measurement. The oils were added to 7 mm wells in the agar after solubilization in 100 µL of 5% DMSO. A well containing 5% DMSO alone served as a control. Plates were incubated at 37 °C for 24 h, and inhibition zones were measured. Each assay was performed in triplicate. Inhibition zones less than 12 mm were considered negative for antibacterial activity [39]. A positive control (the most effective antimicrobial agent) was included to validate the assay

### 4.6. Minimum Inhibitory Concentration (MIC) and Minimum Bactericidal Concentration (MBC) Determination

To determine the minimum inhibitory concentration (MIC) and minimum bactericidal concentration (MBC) values, a broth microdilution assay was performed in 96-well microtiter plates. Bacterial suspensions containing approximately 1 × 10^5^ colony-forming units (CFU) per milliliter were prepared and added to each well. The test compounds and control antibiotics were serially diluted twofold in Luria–Bertani (LB) broth to establish a concentration range of 0.062 to 1024 µg/mL. Plates were incubated at 37 °C for 24 h.

The MIC, defined as the lowest concentration inhibiting visible growth, was determined according to Clinical and Laboratory Standards Institute (CLSI) guidelines. To assess bactericidal activity, colonies from wells exhibiting no visible growth were subcultured onto agar plates and incubated. The MBC was determined as the lowest concentration preventing colony formation, following the method described by Khosravi and Malekan [40].

### 4.7. Ethidium Bromide Efflux Assay

The ethidium bromide (EtBr) efflux assay was performed to evaluate the effects of essential oils on efflux pump activity in *Salmonella* cells. Cells were grown to an OD₆₀₀ of 0.6 in nutrient broth and washed with 20 mM potassium phosphate buffer containing 1 mm MgCl₂. EtBr was added to a final concentration of 5 µM.

Cultures were incubated at 20 °C with shaking for 60 min. After centrifugation, cells were resuspended in buffer containing 5% glucose. Fluorescence was measured over 15 min at excitation and emission wavelengths of 530 and 600 nm using a spectrofluorometer. To evaluate the effects of essential oils, cells were pre-incubated with different concentrations of each oil (0, 250, and 500 µg/mL) for 5 min at 37 °C before the addition of glucose. Higher fluorescence intensity indicates increased EtBr retention within the cells [41].

### 4.8. GC-MS Analysis

GC-MS analysis was conducted using a Shimadzu GC-MS system (Kyoto, Japan) equipped with a gas chromatograph (GC-2010) and a mass spectrometer (MS-QP2010 Plus) at the Central Laboratories Network, National Research Centre, Cairo, Egypt. One milliliter of the sample was diluted with 19 mL of dichloromethane, filtered through a 0.22 µm filter, and then analyzed.

The GC was fitted with a DB-5MS capillary column (30 m × 0.25 mm ID, 0.25 µm film thickness). Helium was used as the carrier gas at a flow rate of 1 mL/min. A 1 µL injection was performed in splitless mode. The GC oven temperature was programmed to start at 60 °C for 2 min, then increase to 280 °C at a rate of 5 °C/min, and hold at 280 °C for 5 min.

The mass spectrometer operated in electron ionization (EI) mode at 70 eV with a scan range of *m*/*z* 40–450. Data acquisition and processing were carried out using Shimadzu GC-MS Solutions software v.4. Compound identification was based on comparing mass spectra with those in the NIST and Wiley spectral libraries.

### 4.9. Molecular Docking Analysis

Molecular docking simulations were performed to assess the potential affinity of the extracted compounds from effective oils against the proximal binding pockets of *Salmonella Typhimurium* MDR efflux pumps AcrAB (Uniprot ID: Q8Z0T3) and AcrD (PDB code: 4R86).

At first, the receptor (protein) structures were downloaded from the Protein Data Bank (PDB), and the receptor structures were cleaned (removing water, ions, and other irrelevant molecules) using PyMOL. Then polar hydrogens were added, and the receptor structures were saved in PDBQT. Additionally, the 2D structure of each compound was drawn using Chem-Bio Draw Ultra16.0, saved as an SDF file, and then converted to a 3D structure. Protonation and energy minimization (0.1 RMSD kcal/mol) were carried out using the MMFF94 force field [42]. The prepared ligands were saved in PDBQT format and docked onto the protein receptors using Autodock Vina 1.5.7 software [43].

The receptor was held rigid while the ligands were allowed to be flexible. During the docking refinement, each molecule was allowed to generate twenty different poses. The docking scores (affinity energy) of the best-fitted poses with the active sites were recorded, and 3D figures were generated using the Discovery Studio 2016 visualizer [44].

### 4.10. Molecular Dynamic (MD) Simulation

Molecular dynamics (MD) simulations were performed using the Desmond simulation package from Schrödinger LLC [45]. The NPT ensemble (heating at 0–300 K. Further, with the time step of 100 ps, the system normalized in an equilibrium state at 1000 steps. The final production run was kept for 100 ns, at the time steps of 100 ps, 300 K temperature and 1.01325 atm pressure, for both complexes applying the Nose-Hoover method with NPT ensemble) was used for all simulations, maintaining a temperature of 300 K and a pressure of 1 bar. The simulations ran for 100 nanoseconds, with a relaxation time of 1 picosecond for the tested ligands. The OPLS_2005 force field parameters were applied throughout the simulations. Long-range electrostatic interactions were calculated using the particle mesh Ewald method, with a cutoff radius of 9.0 Å for Coulomb interactions [46].

Water molecules were explicitly represented using the simple point charge model. Pressure control was maintained using the Martyna–Tuckerman–Klein chain coupling scheme with a coupling constant of 2.0 picoseconds. Temperature control was achieved using the Nosé–Hoover chain coupling scheme. Nonbonded forces were calculated using the r-RESPA integrator. Short-range forces were updated every step, while long-range forces were updated every three steps. Trajectories were saved at 4.8 picosecond intervals for subsequent analysis.

The interactions between ligands and proteins were examined using the simulation interaction diagram tool within the Desmond MD package. The stability of the MD simulations was evaluated by monitoring the root mean square deviation (RMSD) of ligand and protein atom positions over time.

The AMBER 14 package with the AMBER force field ff99 [47] was used for various tasks, including minimization, the addition of counterions, solvation, equilibration, and running periodic box, explicit water (TIP4P) MD simulations for the tested ligands.

The structures of the tested ligands were optimized using the density functional theory B3LYP method with a 6–31G basis set, and parameters were set to the GAFF force field (The B3LYP functional combines Becke’s three-parameter exchange functional with the Lee-Yang-Parr correlation functional. This hybrid function provides a good computational accuracy for molecular systems. Additionally, the optimizations employed the 6–31G(d,p) basis set, which provides a good trade-off between accuracy and computational efficiency for geometry optimization and electronic property evaluation. Moreover, GAFF is designed for small organic molecules and is often used in conjunction with AMBER for hybrid quantum mechanics/molecular mechanics (QM/MM) simulations and molecular dynamics.). The protein–ligand–water system was allowed to be flexible during simulations, which comprised 10 independent runs with different random initial velocities. Each run spanned 10 nanoseconds, utilizing a timestep of 1 femtosecond. Data analysis was conducted using the cpptraj program from the AMBER Tools distribution.

### 4.11. Efflux Pump Gene Detection and Expression Analysis

To investigate the presence and expression of efflux pump genes, conventional and quantitative PCR were performed.

#### 4.11.1. Detection of Efflux Pump Genes by Conventional PCR Assay

Primer sequences for efflux pump genes were designed using Primer3 v.0.4.0 and FastPCR v.6.1 software. Touchdown PCR was employed to optimize primer specificity and sensitivity, with an annealing temperature range of 50–60 °C that decreased by 1 °C per cycle. PCR amplification was carried out in a 25 µL reaction volume containing 12.5 µL of DreamTaq Green PCR Master Mix (2X, Thermo Fisher Scientific, USA), 1 µL of each primer (20 pmol each), 5.5 µL of deionized water, and 5 µL of DNA template. Amplification products were separated on a 1% agarose gel and visualized using GelRed Nucleic Acid Stain. All utilized primer sequences and cycling conditions are demonstrated in Table S1.

#### 4.11.2. Quantitative Analysis of Gene Expression

Total RNA was extracted from both untreated and treated bacterial cultures with sub-inhibitory concentrations of each treatment (defined as concentrations that do not inhibit bacterial growth but can still induce changes in gene expression) using the QIAamp RNeasy Mini Kit (QIAGEN, Hilden, Germany) and quantified using a NanoDrop Eight Spectrophotometer (Thermo Fisher Scientific, Waltham, MA, USA). RNA quality was evaluated based on A260/A280 and A260/A230 ratios, as well as RNA integrity number (RIN) values. Real-time PCR was performed using SYBR Green chemistry on a StepOnePlus Real-Time PCR System (Applied Biosystems, Foster City, CA, USA). The reaction mixture consisted of 10 µL of 2x HERA SYBR^®^ Green RT-qPCR Master Mix (Hera Biolab, Republic of Korea), 1 µL of each primer (20 pmol), 5 µL of RNA, and 3 µL of water. Cycling conditions included an initial denaturation at 95 °C for 15 min, followed by 40 cycles of 95 °C for 15 s, (annealing temperature, T°C, Table 5) for 30 s, and 72 °C for 30 s. A melting curve analysis was performed to confirm PCR product specificity. PCR efficiency for target genes and the reference gene (16S rRNA) was assessed using standard curve analysis. The relative expression levels of target genes were calculated using the 2^−ΔΔCt^ method [48].

### 4.12. Statistical Analysis

Data were analyzed using MS Excel and GraphPad Prism software (version 9.5.0). The normality of data distribution was assessed using the Shapiro-Wilk test, and the homogeneity of variance was evaluated using Levene’s test. To compare the effects of treatments on mRNA gene expression, one-way ANOVA was followed by Tukey’s HSD post hoc test. Data are presented as mean ± standard error (SE). Statistical significance was considered at *p* < 0.05.

## 5. Conclusions

This study presents a groundbreaking discovery of cinnamon and cumin oils as potent inhibitors of the *Salmonella* MDR efflux pumps AcrAB and AcrD. These findings offer a promising avenue for combating the global health threat posed by multidrug-resistant *Salmonella*.

The novelty of this study lies in the comprehensive use of in silico and molecular dynamics simulations to identify and characterize potential inhibitors of these *Salmonella* MDR efflux pumps. Furthermore, the design and optimization of novel primers for the *robA* gene played a crucial role in accurately quantifying efflux pump gene expression. These primers enabled precise measurement of gene expression changes, providing valuable insights into the mechanism of action of the identified inhibitors.

## Figures and Tables

**Table 1:** Serotype distribution, antimicrobial susceptibility, and molecular typing of *Salmonella* isolates.

Isolate No.	Bacterial Serotype	Source	No. of Resistant Antibiotics	No. of Antimicrobial Classes	Resistance Profile	MAR Index	Genotypic Characteristic of Efflux
1	*S. Montevideo*	Stool	2	2	AX, CAZ	0.18	*acrD*, *robA*
2	*S. Montevideo*	Blood	4	3	AX, A/S, GEN, CAZ	0.36	*acrB*, *mdtB*, *acrF*, *acrD*, *robA*
3	*S. Typhimurium*	Blood	4	3	AX, A/S, GEN, CAZ	0.36	*acrB*, *mdtB*, *acrF*, *acrD*, *robA*
4	*S. Typhimurium*	Stool	2	2	AX, CAZ	0.18	*soxS*, *robA*
5	*S. Typhimurium*	Blood	5	4	AX, C, A/S, GEN, CAZ	0.45	*acrB*, *mdtB*, *acrF*, *acrD*, *robA*, *soxS*, *marA*, *mdsB*
6	*S. Anatum*	Stool	5	4	AX, A/S, GEN, OF, CAZ	0.45	*acrB*, *mdtB*, *acrF*, *acrD*, *soxS*, *marA*, *mdsB*, *robA*
7	*S. Anatum*	Stool	5	4	AX, C, A/S, GEN, CAZ	0.45	*acrB*, *mdtB*, *acrF*, *acrD*, *soxS*, *marA*, *mdsB*, *robA*
8	*S. Anatum*	Stool	5	4	AX, C, A/S, GEN, CAZ	0.45	*acrB*, *mdtB*, *acrF*, *acrD*, *soxS*, *marA*, *mdsB*, *robA*
9	*S. Anatum*	Blood	3	3	A/S, GEN, CAZ	0.27	*acrF*, *acrD*, *robA*
10	*S. Typhimurium*	Stool	3	3	A/S, GEN, CAZ	0.27	*marA*, *mdsB*, *robA*
11	*S. Montevideo*	Stool	4	4	C, A/S, GEN, CAZ	0.36	*robA*, *acrB*, *mdtB*, *acrF*, *acrD*
12	*S. Typhimurium*	Stool	3	3	A/S, GEN, CAZ	0.27	*mdtB*, *mdsB*, *robA*
13	*S. Enteritidis*	Minced meat	2	2	AX, CAZ	0.18	Undetermined
14	*S. Typhimurium*	Sausage	4	3	AX, A/S, GEN, CAZ	0.36	*robA*, *acrB*, *mdtB*, *acrF*, *acrD*
15	*S. Enteritidis*	Luncheon	4	3	AX, A/S, GEN, CAZ	0.36	*acrB*, *mdtB*, *acrF*, *acrD*, *soxS*, *marA*, *mdsB*, *robA*
16	*S. Typhimurium*	Luncheon	1	1	CAZ	0.09	Undetermined
17	*S. Enteritidis*	Kofta	6	5	AX, C, A/S, GEN, CAZ, AK	0.55	*acrB*, *mdtB*, *acrF*, *acrD*, *soxS*, *marA*, *mdsB*, *robA*
18	*S. Typhimurium*	Kofta	6	4	AX, A/S, GEN, CAZ, AK, OF	0.55	*acrB*, *mdtB*, *acrF*, *acrD*, *soxS*, *marA*, *mdsB*, *robA*
19	*S. Typhimurium*	Minced meat	6	5	C, A/S, GEN, CAZ, AK, LE	0.55	*acrB*, *mdtB*, *acrF*, *acrD*, *soxS*, *marA*, *mdsB*, *robA*
20	*S. Typhimurium*	Sausage	6	4	AX, C, A/S, GEN, CAZ, AK	0.55	*acrB*, *mdtB*, *acrF*, *acrD*, *soxS*, *marA*, *robA*
21	*S. Enteritidis*	Minced meat	4	3	A/S, GEN, CAZ, AK	0.36	*robA*, *AcrB*, *mdtB*, *acrF*, *acrD*
22	*S. Enteritidis*	Minced meat	3	3	A/S, GEN, CAZ	0.27	*acrD*, *soxS*, *robA*
23	*S. Typhimurium*	Minced meat	4	4	C, A/S, GEN, CAZ	0.36	*acrB*, *mdtB*, *acrF*, *acrD*, *soxS*, *marA*, *mdsB*, *robA*
24	*S. Virchow*	Minced meat	3	3	A/S, GEN, CAZ	0.27	*acrD*, *soxS*, *robA*
25	*S. Virchow*	Minced meat	8	5	C, CIP, A/S, GEN, AK, LE, OF, CAZ	0.73	*acrB*, *mdtB*, *acrF*, *acrD*, *soxS*, *marA*, *mdsB*, *robA*
26	*S. Typhimurium*	Minced meat	5	4	C, CIP, AK, LE, CAZ	0.45	*acrB*, *mdtB*, *acrF*, *acrD*, *soxS*, *marA*, *mdsB*, *robA*
27	*S. Virchow*	Minced meat	7	4	CIP, A/S, GEN, AK, LE, OF, CAZ	0.64	*acrB*, *mdtB*, *acrF, acrD*, *soxS*, *marA*, *mdsB*, *robA*
28	*S. Typhimurium*	Minced meat	6	5	AX, C, A/S, GEN, OF, CAZ	0.55	*acrB*, *mdtB*, *acrF*, *acrD*, *soxS*, *marA*, *mdsB*, *robA*
29	*S. Typhimurium*	Minced meat	6	5	AX, C, A/S, GEN, LE, CAZ	0.55	*acrB*, *mdtB*, *acrF*, *acrD*, *soxS*, *marA*, *mdsB*, *robA*

**Table 2:** Varimax rotated PCA of essential oil antibacterial activity against MDR *Salmonella*.

Essential Oils	PC1	PC2	PC3	PC4
Sage oil	0.434	−0.282	**0.749**	0.281
Nigella oil	−0.375	**0.629**	0.454	−0.210
Ginger oil	**−0.592**	0.562	0.157	0.068
Cumin oil	**−0.787**	−0.384	0.220	−0.279
Cinnamon oil	**−0.742**	−0.458	−0.031	−0.277
Sesame oil	**0.513**	0.472	−0.069	−0.477
Thyme oil	**−0.631**	0.304	−0.196	0.541
Eigenvalue	2.510	1.465	0.885	0.803
Proportion of variance	35.85%	20.93%	12.64%	11.47%
Cumulative proportion of variance	35.85%	56.78%	69.42%	80.89%

Bold loadings are statistically significant.

**Table 3:** Molecular docking analysis of the tested compounds binding to the *Salmonella Typhimurium* MDR efflux pump AcrAB.

Tested Compounds	RMSD Value (Å)	Docking (Affinity) Score (kcal/mol)
Compound **12**	1.42	−6.90
Compound **21**	1.08	−6.69
Compound **22**	1.15	−6.73
Compound **23**	0.87	−6.60
Compound **24**	0.95	−6.98
Compound **31**	1.27	−6.56
Compound **37**	1.51	−5.87
Reference (A1AN8)	0.89	−6.88

**Table 4:** Molecular docking analysis of the tested compounds binding to the *Salmonella Typhimurium* MDR efflux pump AcrD.

Tested Compounds	RMFSD Value (Å)	Docking (Affinity) Score (kcal/mol)
Compound **4**	1.48	−5.56
Compound **12**	0.86	−5.36
Compound **13**	0.95	−6.23
Compound **16**	0.81	−5.61
Compound **18**	1.04	−7.29
Compound **21**	0.93	−6.21
Compound **22**	1.33	−5.41
Compound **23**	1.35	−5.23
Compound **24**	1.21	−6.27
Compound **25**	1.36	−7.64
Compound **29**	1.73	−5.87

**Table 5:** The utilized primers, their sequences, and annealing of target genes for Syper green RT-PCR.

Genes	Primers (5′-3′)	Annealing (T °C)	Reference
*16S rRNA*	F: CCTCAGCACATTGACGTTAC R: CCTCAGCACATTGACGTTAC	60	[49]
*acrB*	F: GACGTCCTATTTTCG R: CGAAGACGCCTCTGT	55	[49]
*acrD*	F: CTGCGCTGGATTCTGATT R: ATAATGGCGAACGAGGAG	55	[49]
*acrF*	F: TCGTGTTGCTAGGCACTT R: GGATCTGCGACATGGATT	55	[49]
*mdtB*	F: TATCGGCTATCGCTTCCT R: TAGAGCGTAACAACCTGAAT	55	[50]
*mdsB*	F: CGATATGTTGATGGTGGTT R: GATGGCGAAGTTAGACAG	55	[50]
*marA*	F: GACCCGGACGTTCAAAAACTAT	55	[51]
	R: TCGCCATGCATATTGGTGAT		
*soxS*	F: CGGAATACACGCGAGAAGGT	55	[51]
	R: GAGCGCCCGATTTTTGATATC		
*robA*	F: CGTTTCGATTCGCAGCAAAC R: GAACTGAACGCGCATCTGAT	60	This study

## Data Availability

All data generated or analyzed during this study are included in this published article and its Supplementary Information Files.

## Supplementary Materials

The following supporting information can be downloaded at: https://www.mdpi.com/article/10.3390/ijms252312949/s1, References [52,53] are cited in supplementary file.
