# Supplementary material for: Natural Inhibitors of Salmonella MDR Efflux Pumps AcrAB and AcrD: An Integrated In Silico, Molecular, and In Vitro Investigation
Source: Int J Mol Sci. 2024 Dec 2;25(23):12949. doi: 10.3390/ijms252312949 (PMC11641668; doi:10.3390/ijms252312949)
Supplement: Supplementary file 1 [file ijms-25-12949-s001.zip › ijms-3306114-supplementary.pdf]

Table S1. The utilized primers and their sequences of target genes for conventional PCR

Genes	Primers (5'-3')	Product size	Annealing (T°C)	Reference
16S rRNA	F: TTCTTCATACACGCGGCATG	201 bp	58°C	This study
	R: TTCTTCATACACGCGGCATG			
acrB	F: CGGTATTGCATTCGTGTCGT	242 bp	60°C	This study
	R: CGCCGAACAACTGATTACGT			
acrD	F: AATTGTGCGTGAAGCGGT	100 bp	58°C	[1]
	R: GCTACAGCGCCATAGTAA			
acrF	F: GAGCTTGGCGGTGAAAACTA	653 bp	58°C	[2]
	R: AACGCAACCAGAACGGATAG			
mdtB	F: CCGAATCCGCCGATTTACAG	803 bp	60°C	[2]
	R: GCCATCAGCGTCAGGTTATT			
mdsB	F: ATCCAAAGTCCGGGTGCTAA	192 bp	60°C	This study
	R: CACGACAAGAACAACCAGCA			
marA	F: ACCGGTCATTCATTAGGCCA	164 bp	60°C	This study
	R: GGCGGCACATCAAAGTAGTT			
soxS	F: ATACACGCGAGAAGGTTTGC	170bp	50°C	This study
	R: GTGGTATTTGCAGCGGATGT			
robA	F: CGTTTCGATTCGCAGCAAAC	249bp	60°C	This study
	R: GAACTGAACGCGCATCTGAT			

Table S2. SIC, MIC, and MBC of cinnamon and cumin oils against S*almonella* isolates

Isolate no.	Concentration of SIC, MIC, MBC (µg/ml)					
	Cinnamon oil extract			Cumin oil extract		
	SIC	MIC	MBC	SIC	MIC	MBC
1	2	4	8	0.125	0.25	0.5
2	0.25	0.5	1	4	8	16
3	2	4	8	2	4	8
4	8	16	32	0.25	0.5	1.0
5	1	2	4	2	4	8
6	8	16	32	2	4	8
7	4	8	16	16	32	64
8	8	16	32	0.5	1	2
9	0.25	0.5	1	4	8	16
10	2	4	8	4	8	16
11	0.25	0.5	1	2	4	8
12	0.25	0.5	1	0.5	1	2
13	0.25	0.5	1	0.5	1	2
14	0.25	0.5	1	0.5	1	2
15	0.25	0.5	1	0.5	1	2
16	0.25	0.5	1	2	4	8
17	0.25	0.5	1	0.125	0.25	0.5
18	8	16	32	0.25	0.5	1.0
19	1	2	4	2	4	8
20	8	16	32	2	4	8
21	4	8	16	4	8	16
22	0.25	0.5	1.0	0.5	1	2
23	0.25	0.5	1.0	0.5	1	2
24	0.125	0.25	0.5	0.5	1	2

25	0.125	0.25	0.5	0.5	1	2
26	0.125	0.25	0.5	0.25	0.5	1
27	0.125	0.25	0.5	4	8	16
28	0.125	0.25	0.5	2	4	8
29	0.125	0.25	0.5	0.25	0.5	1

Table S3. GC-MS analysis of predominant bioactive compounds of cinnamon oil extract

Peak	RT	Compound name	Formula	Area%
1	10.521	Benzaldehyde	C7H6O	0.18
2	12.602	1,6-Heptadiyne	C7H8	0.01
3	12.864	Benzyl alcohol	C7H8O	71.51
4	13.149	Formic acid, phenylmethyl ester	C8H8O2	0.05

RT: Retention time, Area %: Relative concentrations

Table S4. GC-MS analysis of predominant bioactive compounds of cumin oil extract

Peak	RT	Compound name	Formula	Area
1	8.64	(1S)-2,6,6-Trimethylbicyclo[3.1.1]hept-2-ene	C10H16	0.27
2	9.959	beta.-Pinene	C10H16	16.86
3	10.606	alpha.-Phellandrene	C10H16	1.13
4	10.921	P-Cymene Cyclohexene, 4-methyl-3-(1-methylethylidene)-	C10H16	0.19
5	11.168	Cyclohexene, 4-methyl-3-(1-methylethylidene)-	C10H16	0.3
6	11.273	Limonene	C10H16	7.94
7	11.411	o-Cymene	C10H14	0.18
8	11.635	Eucalyptol	C10H18O	0.01
9	11.873	1,9-Decadiyne	C10H14	39.74
10	12.887	gamma.-Terpinene	C10H16	0.07
11	15.597	3-p-Menthen-7-al	C10H16O	2.21
29	17.84	2-Caren-10-al	C10H14O	100

RT: Retention time, Area %: Relative concentrations

Table S5. IUPAC/Common name of components

NO	IUPAC/Common name
1	Benzaldehyde
2	hepta-1,6-diyne
3	Phenylmethanol
4	benzyl formate
5	Cinnamaldehyde
6	(1S)-2,6,6-trimethylbicyclo[3.1.1]hept-2-ene
7	6,6-dimethyl-2-methylenebicyclo[3.1.1]heptane
8	5-isopropyl-2-methylcyclohexa-1,3-diene
9	4-methyl-3-(propan-2-ylidene)cyclohex-1-ene
10	1-methyl-4-(prop-1-en-2-yl)cyclohex-1-ene
11	o-cymene
12	(1s,4s)-1,3,3-trimethyl-2-oxabicyclo[2.2.2]octane
13	deca-1,9-diyne
14	1-isopropyl-4-methylcyclohexa-1,4-diene
15	1-methylpyrrolidin-2-one
16	8,8-dimethyl-6,7-diazatricyclo[3.2.1.02,4]oct-6-ene
17	(E)-deca-1,5,9-triene
18	2-nitrodec-1-en-4-yne
19	4-(2-methylenecyclopropyl)butanal
20	3,7-dimethylocta-1,6-diene
21	non-2-yn-1-ol
22	1-isopropyl-4-methylcyclohex-3-en-1-ol
23	4-isopropylcyclohex-3-ene-1-carbaldehyde
24	2-methyl-1-(p-tolyl)propan-2-ol
25	(1S,3R,5R)-1-isopropyl-4-methylenebicyclo[3.1.0]hexan-3-ol
26	(1R,2R,5R)-4,6,6-trimethylbicyclo[3.1.1]hept-3-en-2-yl acetate
27	4-isopropylbenzaldehyde
28	2,2-dimethyl-3-methylenebicyclo[2.2.1]heptane
29	1-allyl-4-methoxybenzene
30	4-isopropylcyclohex-3-ene-1-carbaldehyde
31	7,7-dimethylbicyclo[4.1.0]hept-2-ene-3-carbaldehyde
32	(4-isopropylphenyl)methanol
33	(4-isopropylcyclohexa-1,4-dien-1-yl)methanol
34	1-methyl-2-(prop-1-en-2-yl)-4-(propan-2-ylidene)-1-vinylcyclohexane
35	dodeca-1,11-diyne
36	(1R,9S,Z)-4,11,11-trimethyl-8-methylenebicyclo[7.2.0]undec-4-ene
37	(E)-7,11-dimethyl-3-methylenedodeca-1,6,10-triene

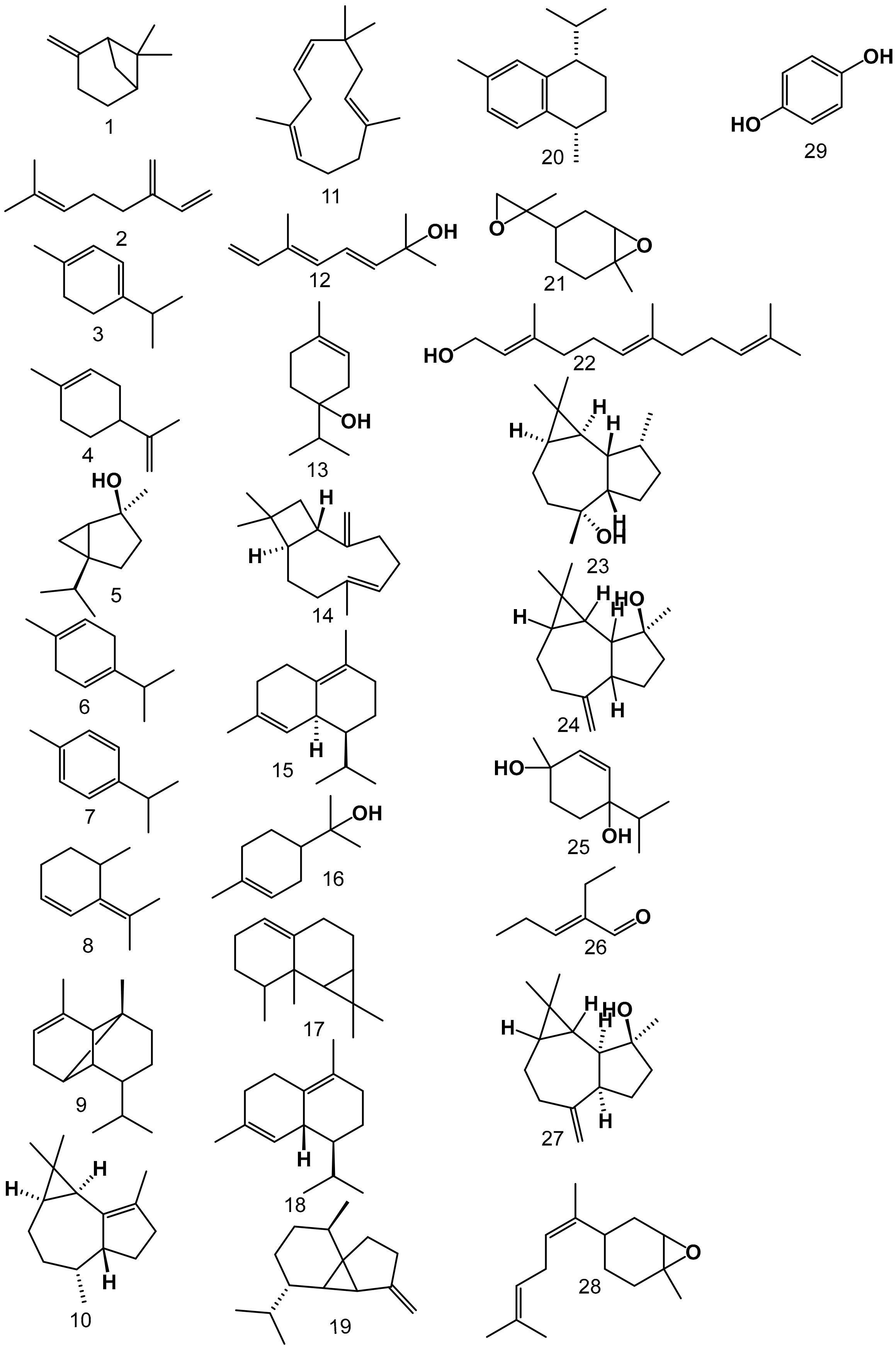
1
2
3
4
5
6
7
8
9
10
11
12
13
14
15
16
17
18
19
20
21
22
23
24
25
26
27
28
29

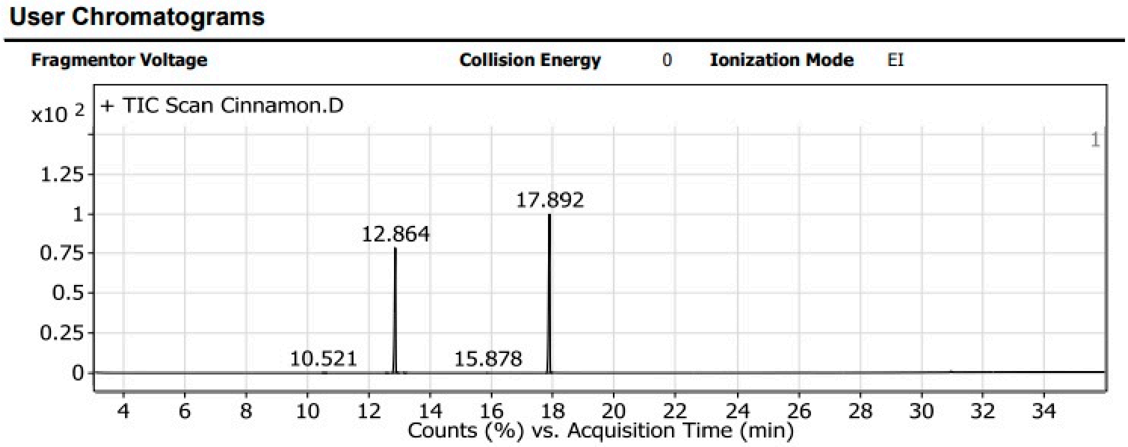

Figure S1. GC chromatography photo of cinnamon oil extract

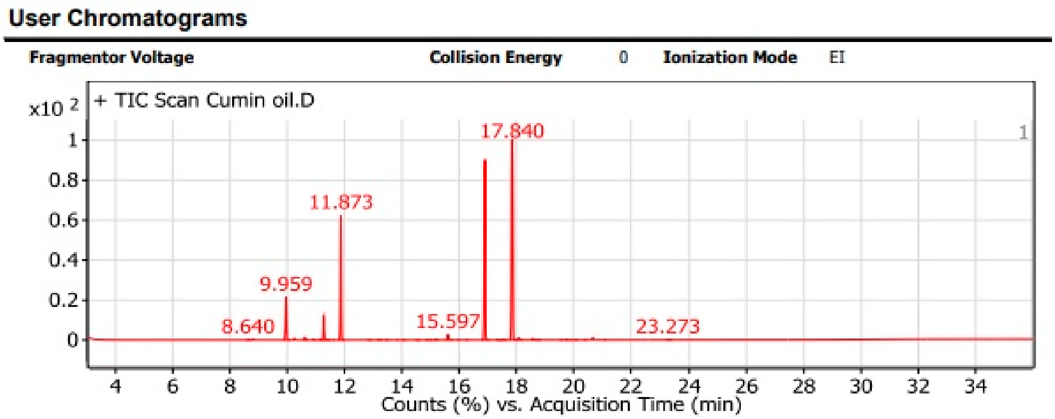

Figure S2. GC chromatography photo of cumin oil extract

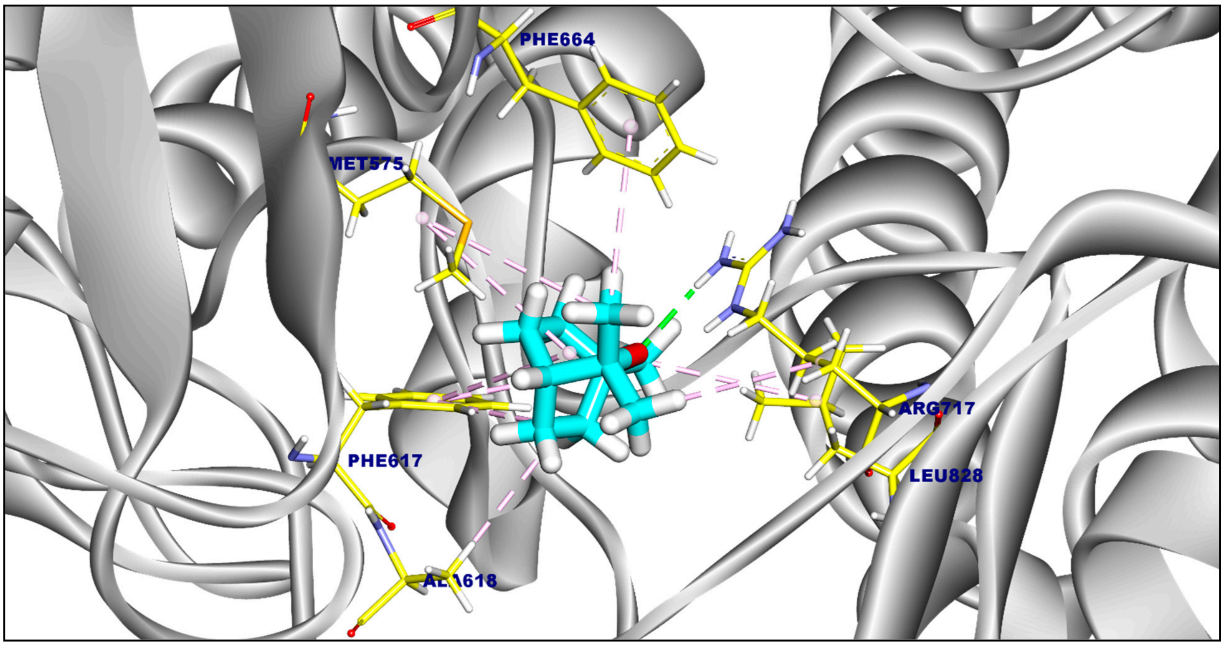

Figure S3. 3D figure of Compound 12 against Salmonella MDR efflux pump acrAB .

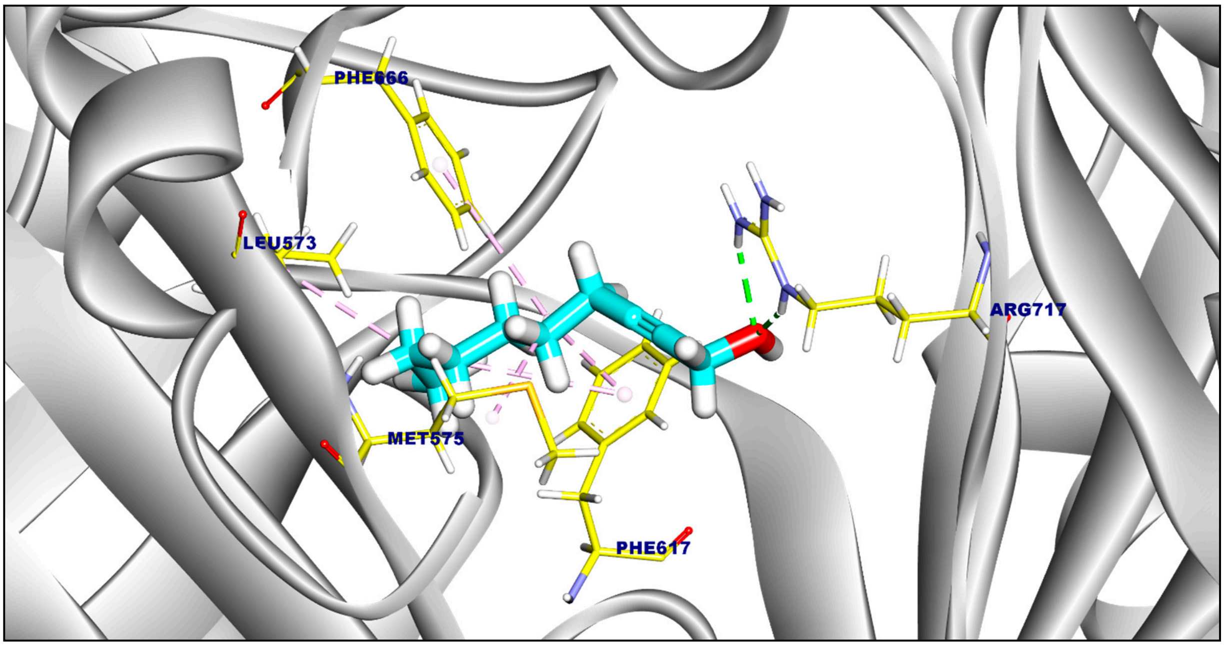

Figure S4. 3D figure of Compound 21 against Salmonella MDR efflux pump acrAB.

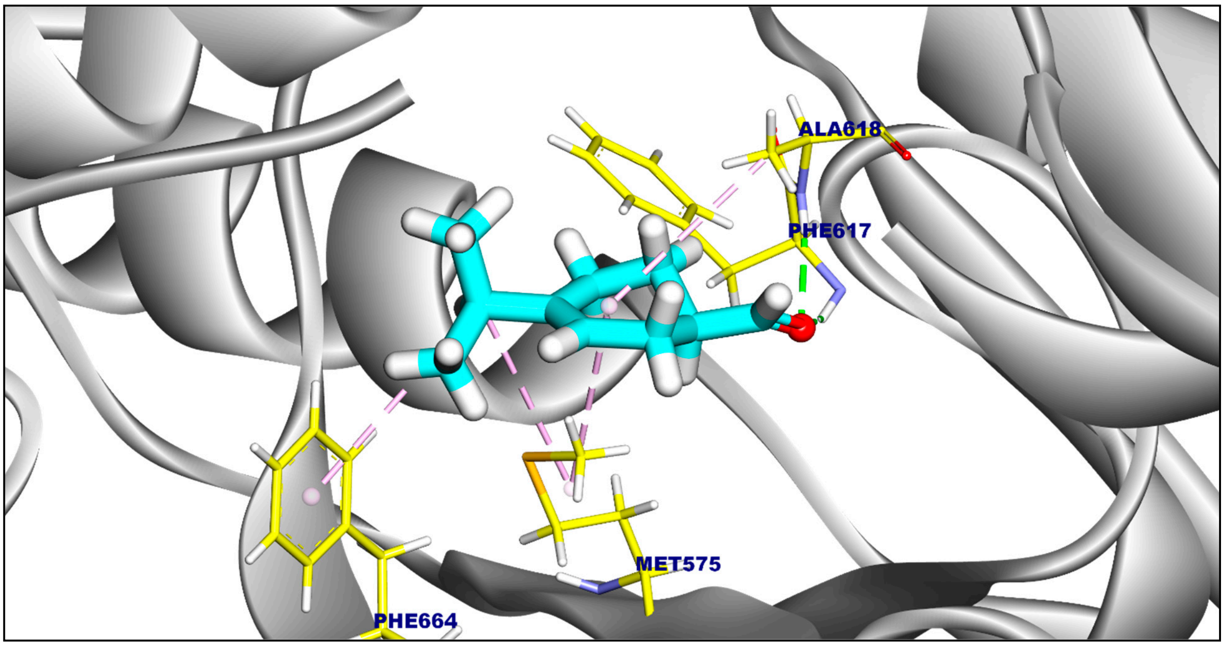

Figure S5. 3D figure of Compound 23 against Salmonella MDR efflux pump acrAB.

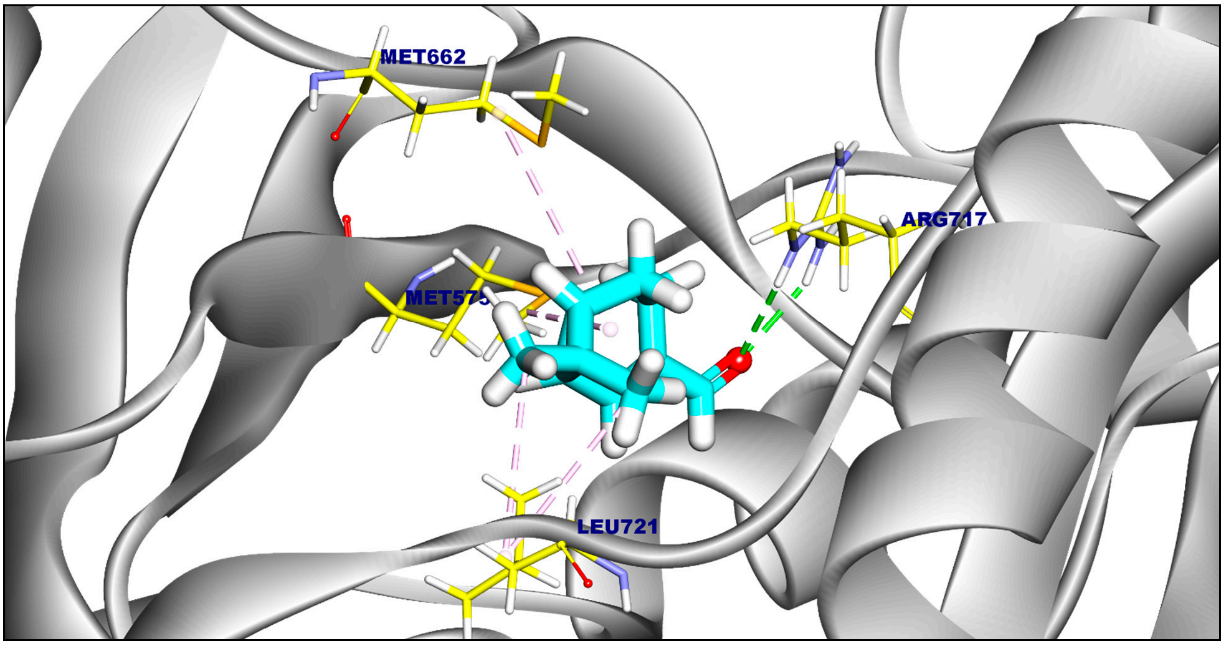

Figure S6. 3D figure of **Compound 31** against **Salmonella MDR efflux pump acrAB**.

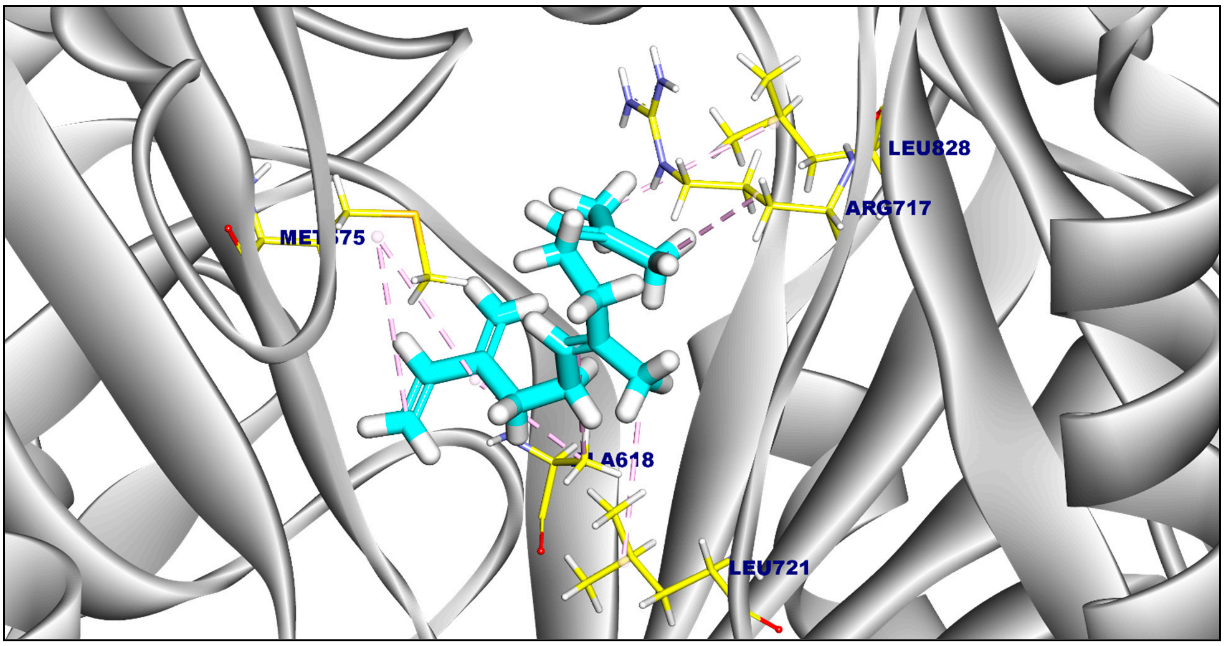

Figure S7. 3D figure of **Compound** 37 against **Salmonella MDR efflux pump acrAB**.

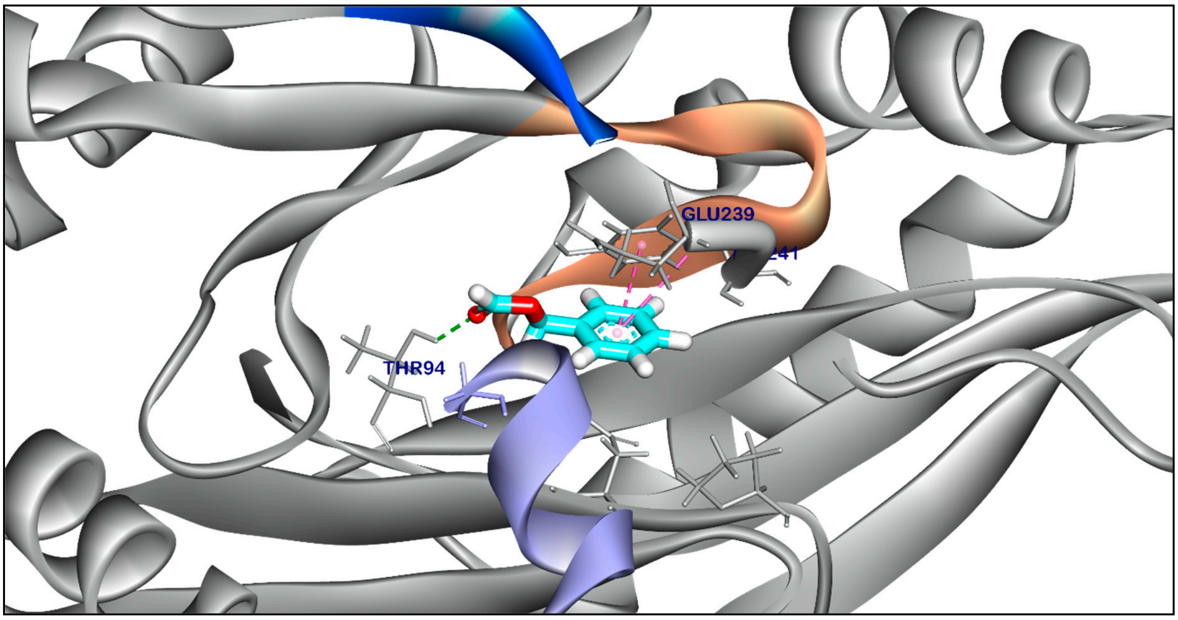

Figure S8. 3D orientation and of Compound 4 against Salmonella efflux pump target site.

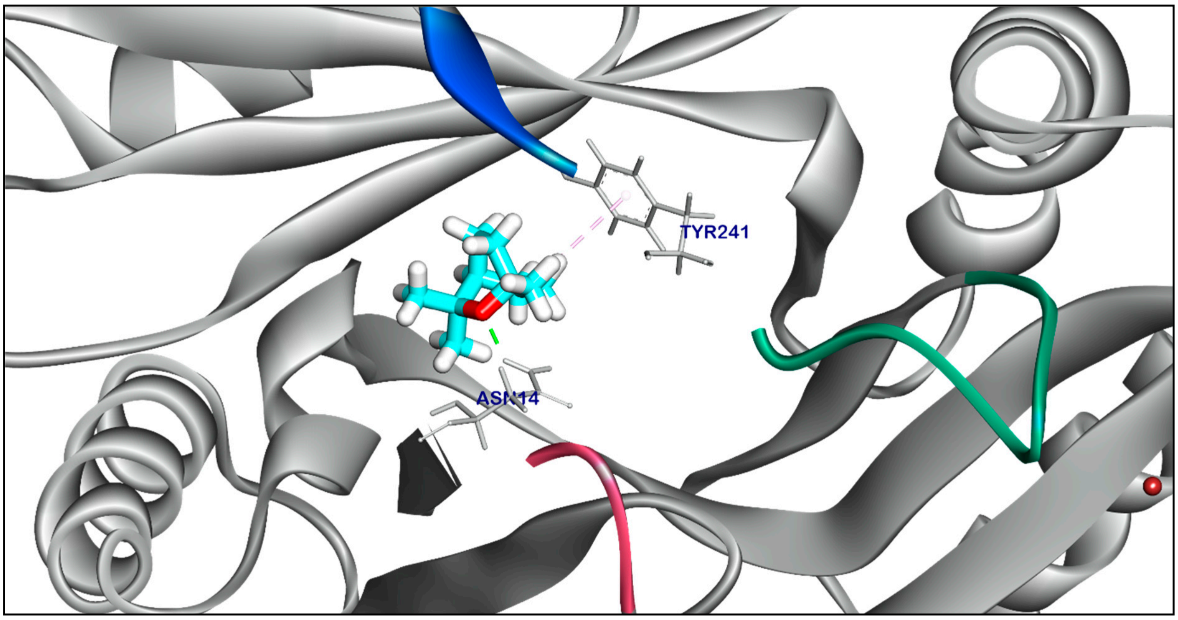

Figure S9. 3D orientation and of Compound 12 against Salmonella efflux pump target site.

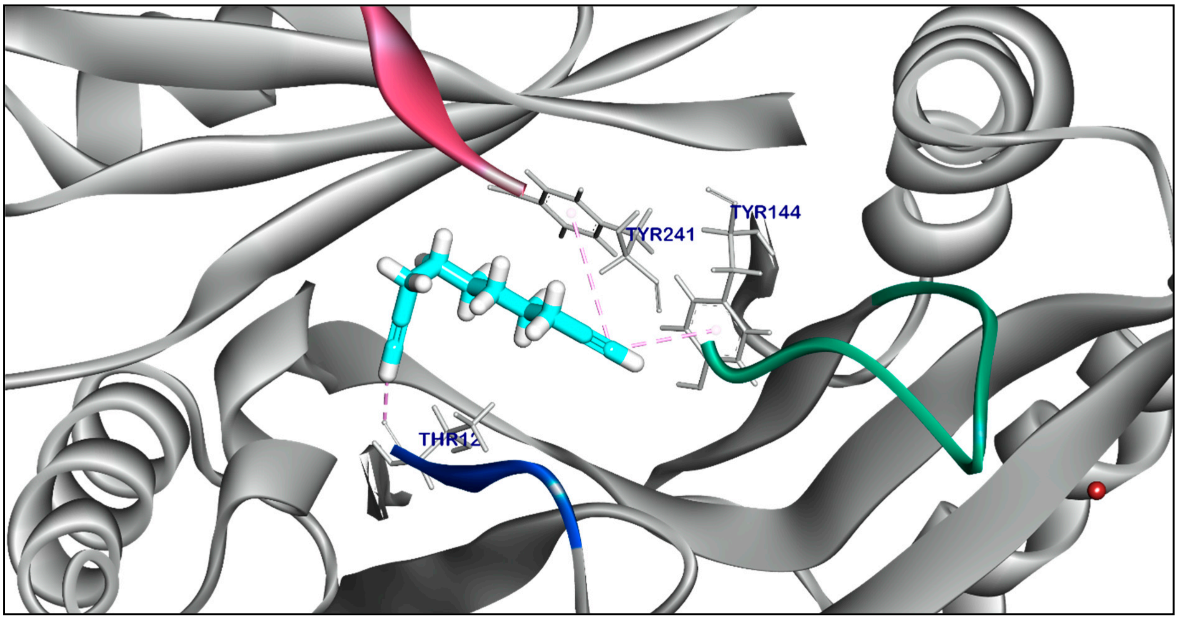

Figure S10. 3D orientation and of Compound 13 against Salmonella efflux pump target site.

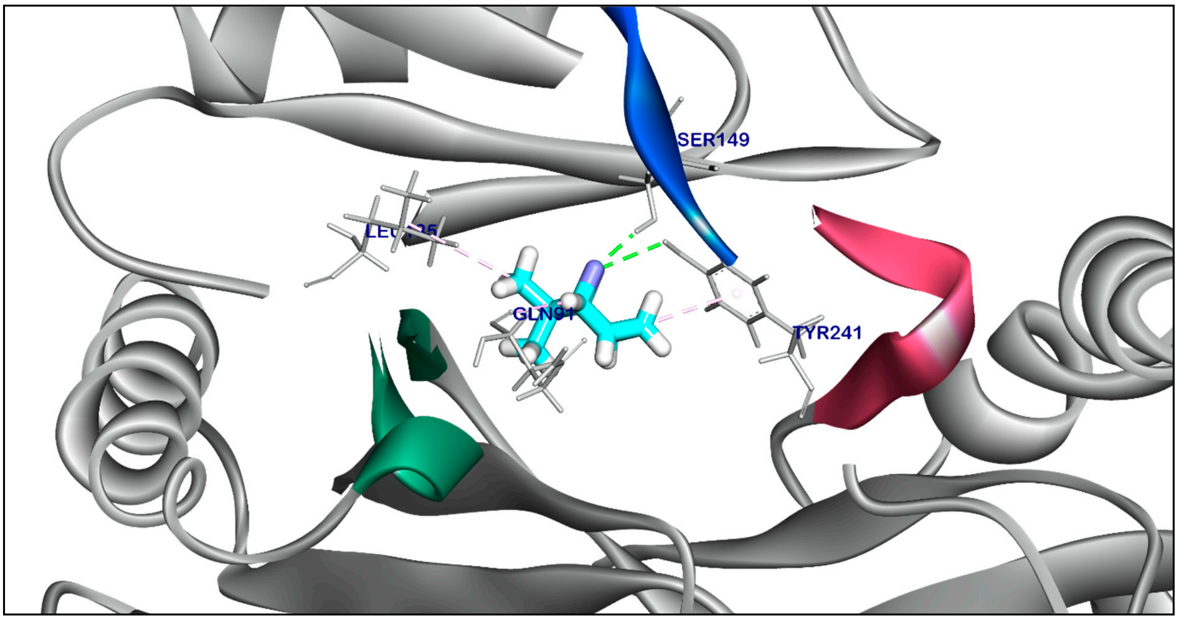

Figure S11. 3D orientation and of Compound 16 against Salmonella efflux pump target site.

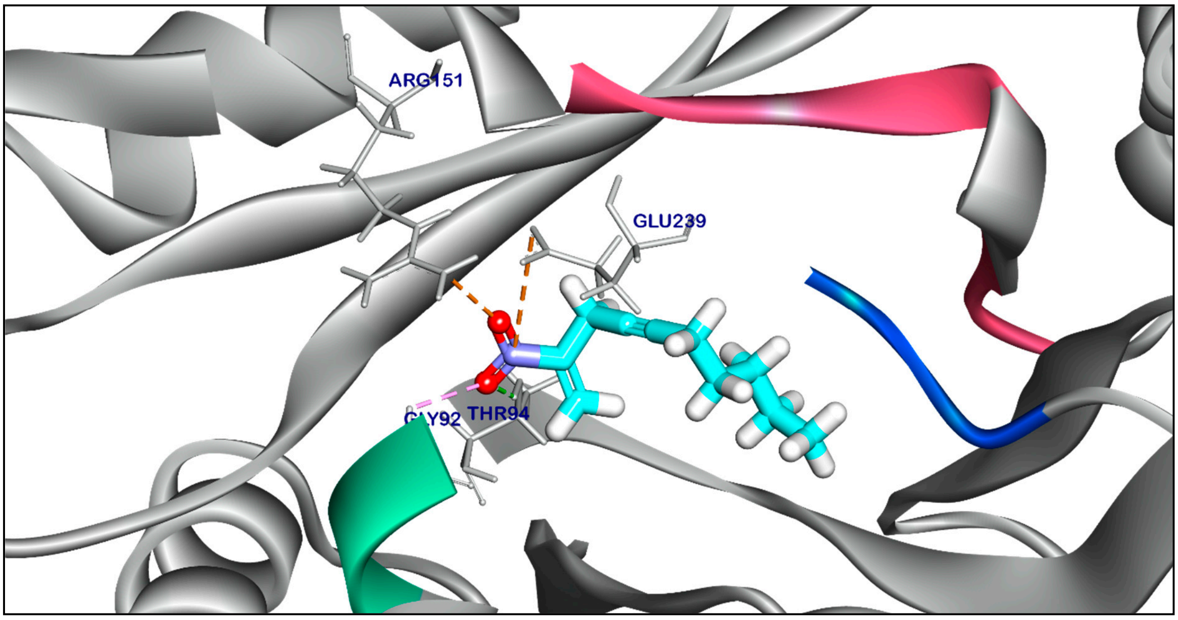

Figure S12. 3D orientation and of Compound 18 against Salmonella efflux pump target site.

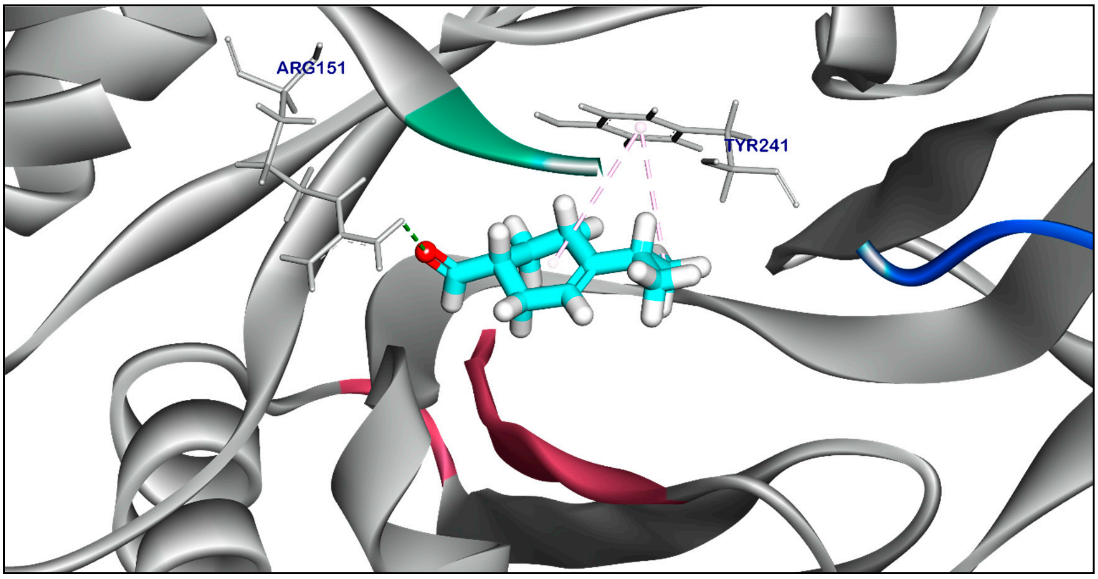

Figure S13. 3D orientation of Compound 23 against Salmonella efflux pump target site.

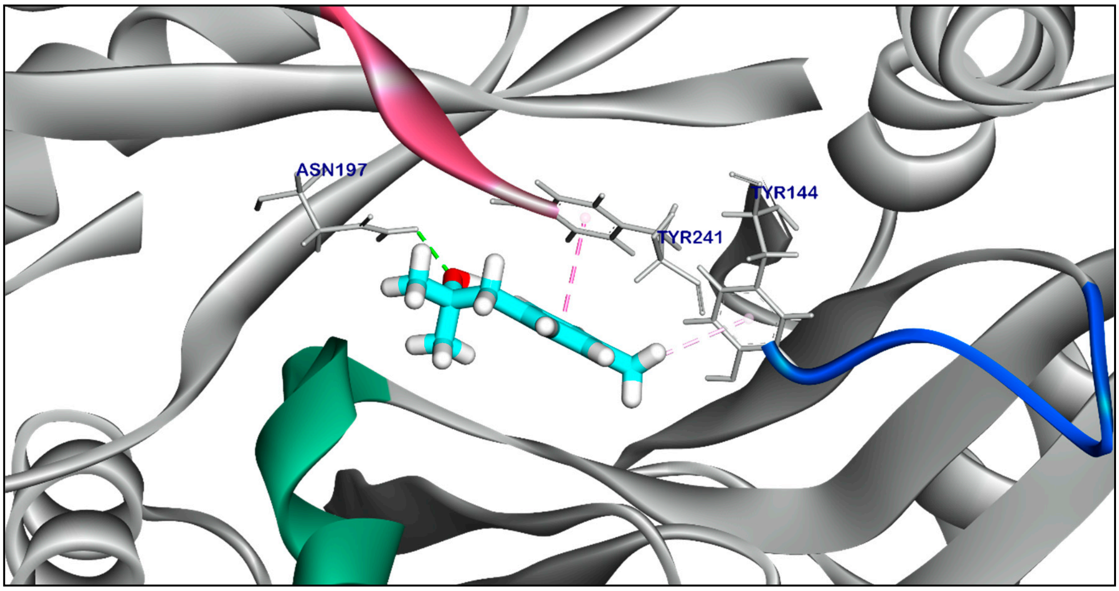

Figure S14. 3D orientation and of Compound 24 against Salmonella efflux pump target site.

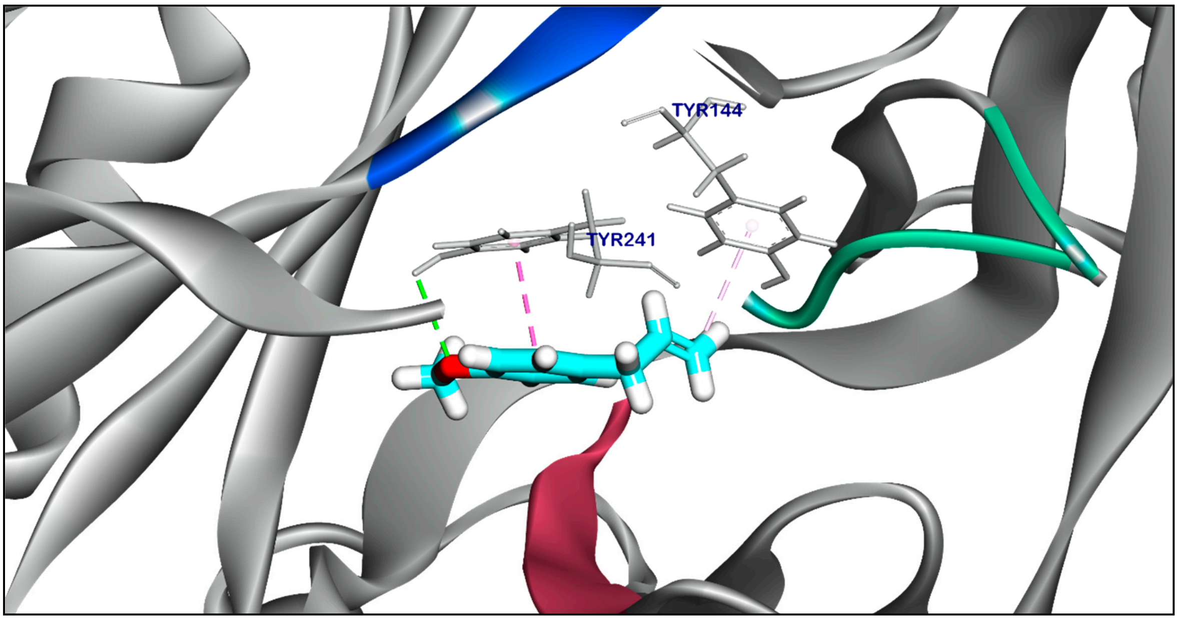

Figure S15. 3D figure of Compound 29 against Salmonella efflux pump target site.

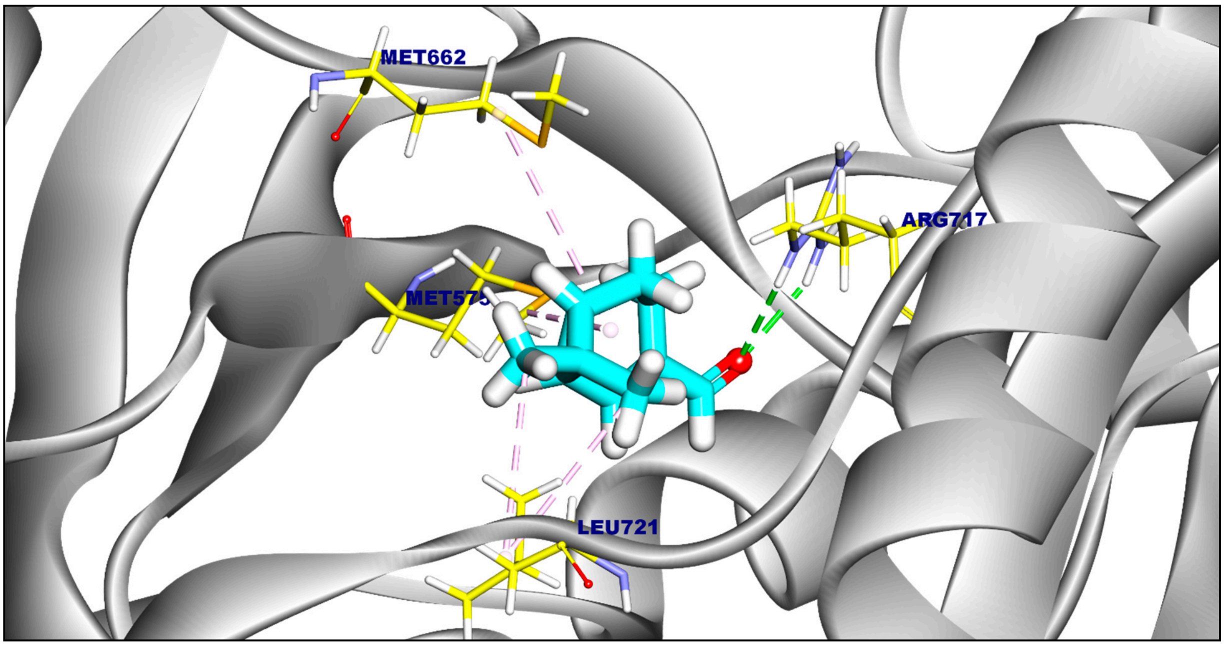

Figure S16. 3D figure of Compound 31 against Salmonella MDR efflux pump acrAB.

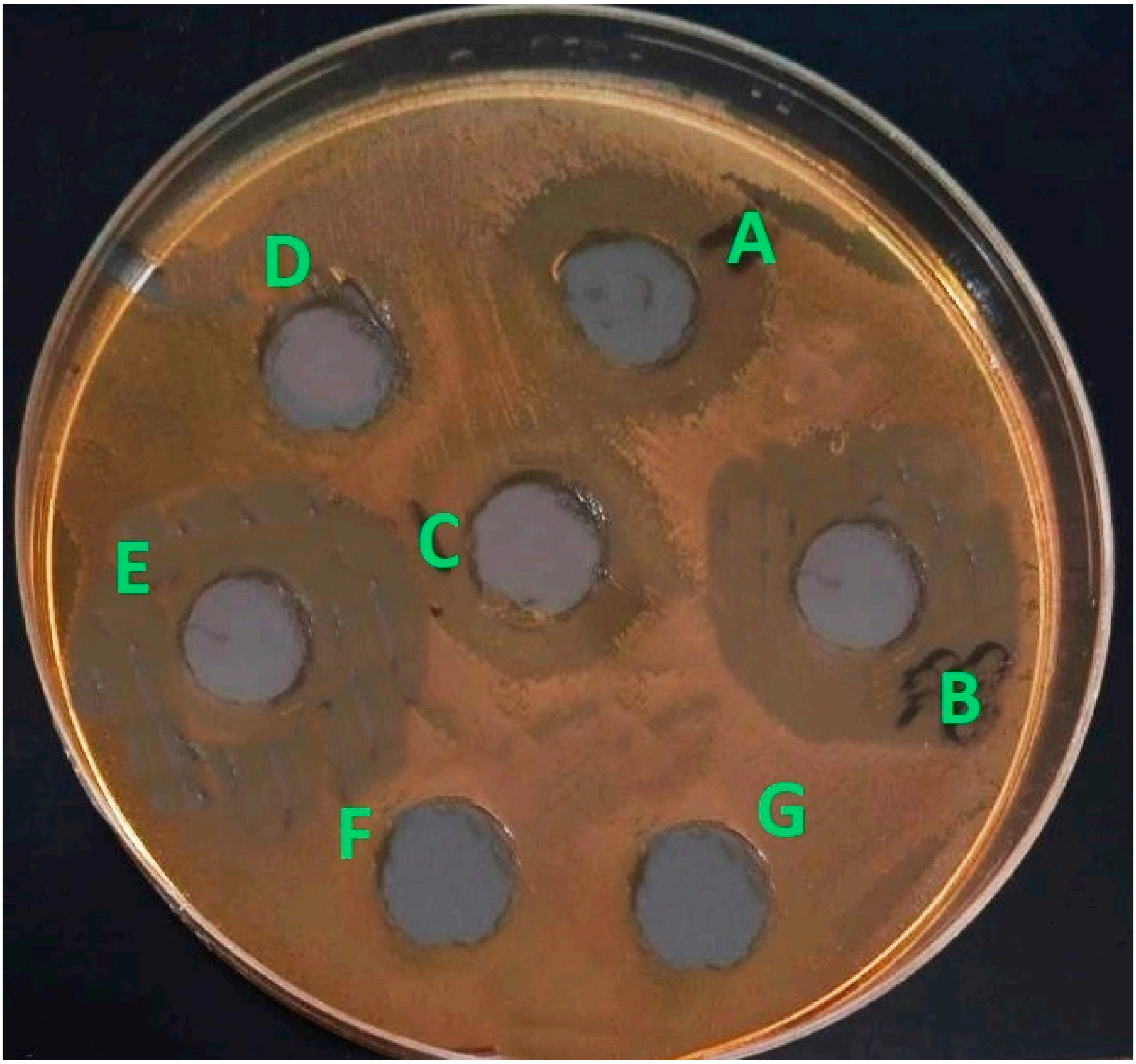

Figure S17. Agar well diffusion assay shows the inhibition zones of Salmonella isolate (code no. 28) against tested seven oil extracts; A, Ginger; B, Cumin; C, Thyme; D, Nigella sativa; E, Cinnamon; F, Sega; G, Sesame

References

1. Baugh, S. The Role of Multidrug Efflux Pumps in Biofilm Formation of Salmonella Enterica Serovar Typhimurium, University of Birmingham, 2014.

2. Chen, S.; Cui, S.; McDermott, P.F.; Zhao, S.; White, D.G.; Paulsen, I.; Meng, J. Contribution of Target Gene Mutations and Efflux to Decreased Susceptibility of Salmonella Enterica Serovar Typhimurium to Fluoroquinolones and Other Antimicrobials. Antimicrob Agents Chemother 2007, 51, 535–542.
